# The Etiology of Multiple Sclerosis: Genetic Evidence for the Involvement of the Human Endogenous Retrovirus HERV-Fc1

**DOI:** 10.1371/journal.pone.0016652

**Published:** 2011-02-02

**Authors:** Bjørn A. Nexø, Tove Christensen, Jette Frederiksen, Anné Møller-Larsen, Annette B. Oturai, Palle Villesen, Bettina Hansen, Kari K. Nissen, Magdalena J. Laska, Trine S. Petersen, Sandra Bonnesen, Anne Hedemand, Tingting Wu, Xinjie Wang, Xiuqing Zhang, Tomasz Brudek, Romana Maric, Helle B. Søndergaard, Finn Sellebjerg, Klaus Brusgaard, Anders L. Kjeldbjerg, Henrik B. Rasmussen, Anders L. Nielsen, Mette Nyegaard, Thor Petersen, Anders D. Børglum, Finn S. Pedersen

**Affiliations:** 1 Department of Human Genetics, University of Aarhus, Aarhus C, Denmark; 2 Department of Medical Microbiology, University of Aarhus, Aarhus C, Denmark; 3 Department of Neurology, Glostrup Hospital, Glostrup, Denmark; 4 Department of Neurology, The Danish Multiple Sclerosis Research Center, Copenhagen University Hospital, Copenhagen, Denmark; 5 Bioinformatics Research Center, University of Aarhus, Aarhus C, Denmark; 6 Department of Neurology, Aarhus University Hospital, Aarhus C, Denmark; 7 BGI-Shenzhen, Shenzhen, China; 8 Department of Clinical Genetics, Odense University Hospital, Odense, Denmark; 9 Department of Molecular Biology, University of Aarhus, Aarhus C, Denmark; 10 Research Institute of Biological Psychiatry, Psychiatry Center Sct. Hans, Roskilde, Denmark; University of Oxford, United Kingdom

## Abstract

We have investigated the role of human endogenous retroviruses in multiple sclerosis by analyzing the DNA of patients and controls in 4 cohorts for associations between multiple sclerosis and polymorphisms near viral restriction genes or near endogenous retroviral loci with one or more intact or almost-intact genes. We found that SNPs in the gene *TRIM5* were inversely correlated with disease. Conversely, SNPs around one retroviral locus, HERV-Fc1, showed a highly significant association with disease. The latter association was limited to a narrow region that contains no other known genes. We conclude that HERV-Fc1 and TRIM5 play a role in the etiology of multiple sclerosis. If these results are confirmed, they point to new modes of treatment for multiple sclerosis.

## Introduction

Two views have dominated the discussions on the etiology of multiple sclerosis (MS[MIM 126200]) in recent decades: It could be a genetic disease, or it could be a disease caused by an infectious agent. The genetic view is most clearly expounded by studies of twins, showing that concordance among monozygotic twins is approximately 25 percent, while concordance among dizygotic twins is only 2–3 percent [1]. The alternative view, that multiple sclerosis is caused by an infectious agent, is most clearly backed by animal studies [2]–[4]. The obvious intersections of a genetic and a viral etiology are the human endogenous retroviruses, which are Mendelian inherited proviral loci in the human genome.

The endogenous human retroviruses exist in multiple types, generally named after the amino acid of the tRNA that serves as primer for first strand synthesis. Some endogenous virus types (−H and −W) are old and universal in a large range of species, whereas certain of the −K type integrations are recent and still segregate in the human population. HERV-Fc endogenous viruses can be traced as far back as the simian forefather, however present copies in our genome are more recent acquisitions and specifically the HERV-Fc1 locus on the X-chromosome, with which this paper is concerned, is shared by man, chimpanzee, and gorilla, but not orangutan. This put the time of integration at about 10 to 15 million years ago [5]. We have confirmed the absence of HERV-Fc1 in orangutan and African Green Monkey by specific PCR (Magdalena J. Laska, unpublished). Homology studies of the *pol* genes have shown that HERV-Fc is closely related to HERV-H, more distantly related to HERV-W and only remotely related to HERV-K [6].

In this study, we have attempted to evaluate the role of retroviruses in the etiology of Multiple Sclerosis, both indirectly by studying genes that restrict retroviruses, and directly by searching for an endogenous retrovirus associated with occurrence of disease.

## Results

Inspired by the classical genetic studies of the mouse restriction gene for retroviruses, *Fv-1* [7], we first attempted to evaluate the importance of retroviruses for MS indirectly. Several human restrictions genes are known to influence retroviral replication to various degrees. If MS were caused by a retrovirus, these genes could well influence the risk of disease. Of the restriction genes, *TRIM5* appears to be the closest functional relative of *Fv-1* [8]. Therefore, to substantiate the involvement of a retrovirus in multiple sclerosis we sought for linkage disequilibrium between markers in *TRIM5* and disease. Initially, we investigated a small cohort consisting of 28 verified cases [9] and 24 controls. We tested 12 SNPs, located in *TRIM5* intron 1. The nearby exon 2 encodes the RING domain in the N-terminus of the protein. This domain plays a major role in recognition of retroviral cores. The SNPs and their primers are listed in Table S1. We found that 7 of the SNPs were suggestive of disequilibrium between SNP and disease (p≤0.1) (Table S2). Table 1 shows the results with the best of these, rs3802981, giving a p-value of 0.004 for association. After Bonferroni correction the p value became 0.05, i.e. the results were just globally significant. The table also shows the results from the analysis of another larger cohort (Cohort2). This cohort consisted of DNA from 350 patients with verified multiple sclerosis [9] living in Western Denmark as well as DNA from 500 persons without sclerosis from the same part of the country. Females constituted 62 percent of cases and 67 percent of the controls. Again, rs3802981 was associated with disease (p = 0.008). The combined p-value for the two cohorts using Fisher's formula was 0.0003; after Bonferroni correction the p value was 0.004.

**Table 1:** Association of multiple sclerosis with the polymorphism rs3802981 located in the first intron of *TRIM5*.

Cohort	Status	CC	CT	TT	Fraction of TT genotypes	OR (CI95%) CC + CT vs TT	P-chitest (Pearson)
1	Cases	2	12	14	0.50	0.20 (0.05–0.74)	0.004
	Controls	10	10	4	0.17		
2	Cases	76	154	114	0.49	0.64 (0.47–0.86)	0.008
	Controls	122	292	131	0.31		
1+2	Cases	78	166	128	0.52	0.59 (0.44–0.79)	0.002
	Controls	132	302	135	0.31		F: 0.0003

F: p-value as calculated from cohort1 and 2 by Fisher's formula for the combination of p-values.

We were similarly inspired by the classical Mendelian studies of the highly active endogenous ecotropic viruses of the AKR mouse [10], [11], which showed that genetic mapping of germ line retroviruses is straightforward. Therefore, we attempted to associate multiple sclerosis with specific human endogenous retroviruses by genetic epidemiology. Intact or near-intact human endogenous retroviruses were treated as inheritable loci in the analyses. We speculated that in order to contribute to disease a retroviral sequence should contain at least one functional or near-functional major gene i.e. a gene with at most 2 stop-codons or frame-shifts, and excluded viruses that did not fulfill this criterion using the RetroSearch tool [12]. Moreover, while many such retroviruses are rather similar, their sites of integration are generally unique, so by testing neighboring SNPs (less that 10 kb away from the virus) we should be able to test the viral loci individually. A cartoon of the location of all such endogenous viruses in the human genome can be found in Figure S1. A list of SNPs near these viruses, for which we could perform genotyping, is shown in Table S3, sorted into 13 plexes for the Sequenom and shown together with the primers. Of the 52 retroviruses identified as relevant 48 were covered by this approach, while 4 could not be tested for association.

Initially, we investigated 193 SNPs in the DNA from Cohort 2. There were clear-cut clusters of significant SNPs in a few chromosomal locations. These locations were substantiated by analyzing additional nearby SNPs. A total of 220 SNPs were tested. The most significant results are indicated in Figure S1, and all results are listed in Table S4.

A particularly striking cluster of significant SNPs occurred on chromosome X at approximate chromosome position 97100000 around the HERV-Fc1 proviral locus (NCBI genome build 37.1). Here, 3 close-lying SNPs gave p-values less than 0.001. The SNP rs391745 was lowest with a p-value of 4*10^−6^ (2-sided) for association with disease, when calculating on the basis of C-allele carriers (Table 2, cohort 2). After Bonferroni correction this p-value was 0.001.

**Table 2:** Association of multiple sclerosis with rs391745 located near HERV-Fc1.

Cohort	Status	C-allele carriers	Non-carriers	Fraction C-allele	OR (CI95)	P (2-sided)
				carriers	for carriers	
2	Cases	79	246	0.24	2.29 (1.60–3.28)	4*10^−6^
	Controls	68	485	0.12		
3	Cases	99	407	0.20	1.43 (1.09–1.89)	0.010
	Controls	165	972	0.15		
4	Cases	35	196	0.15	1.01 (0.64–1.59)	0.97
	Controls	59	333	0.15		
2+3+4	Cases	213	849	0.20	1.54 (1.27–1.87)	1.3*10^−5^
	Controls	292	1790	0.14		F: 6.4*10^−6^

The p-values were calculated from the numbers of carriers and non-carriers using Pearson's Chi-square test. The p-value for all 3 groups marked F was calculated from the individual p-values using Fisher's formula.

We retested the association of rs391745 and multiple sclerosis in Cohort 3. This cohort consisted of 542 cases and 1165 controls with 67 percent female cases and 65 percent female controls. rs391745 was again associated with MS (p = 0.01) (Table 2, cohort 3). Finally, we tested a third, smaller cohort of verified multiple sclerosis consisting of 235 cases and 407 controls in Eastern Danes (Cohort 4). In this cohort women constituted 70 and 63 percent among cases and controls, respectively. We could not find any association of rs391745 with multiple sclerosis in this cohort (p = 0.97) (Table 2, cohort 4), and do not know, why the association was absent. The p-value for all 3 cohorts combined was 0.00002 (2-sided) and the p-value was thus significant after Bonferroni correction (p = 0.004). Similar conclusions were reached when the association was calculated on the basis of allele frequencies, taking into account that males are hemizygous for the SNP (Table S5). In this case, Bonferroni-analysis showed that the corrected p-value was 0.0076. The female controls of cohort 3 were in marginal Hardy-Weinberg disequilibrium (p = 0.067), the females in the other cohorts were in HW equilibrium (p>0.1).

**Figure 1:**
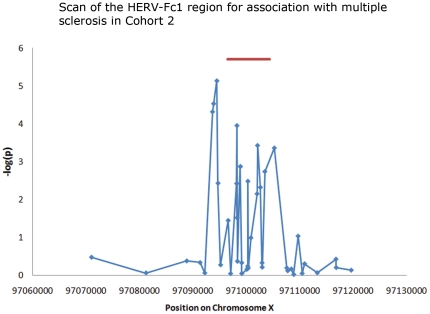
A scan of polymorphisms in the region around HERV-Fc1 for association with multiple sclerosis. The provirus stretches from 97096500 to 97104400 and is marked in red.

To substantiate the association of HERV-Fc1 with multiple sclerosis, we performed a scan of the region surrounding the HERV-Fc1 provirus in Cohort 2. Table S3, plex 11–13 includes the extra SNPs tested. Figure 1 shows the association of a number of polymorphisms in and around the locus in relation to their position on the chromosome. It is clear that the association occurred in a very narrow region around the provirus. In contrast, the nearest known genes lie 141 kb upstream, and 57 kb downstream, respectively. No microRNA genes or small nucleolar RNA genes have been reported in the region.

**Figure 2:**
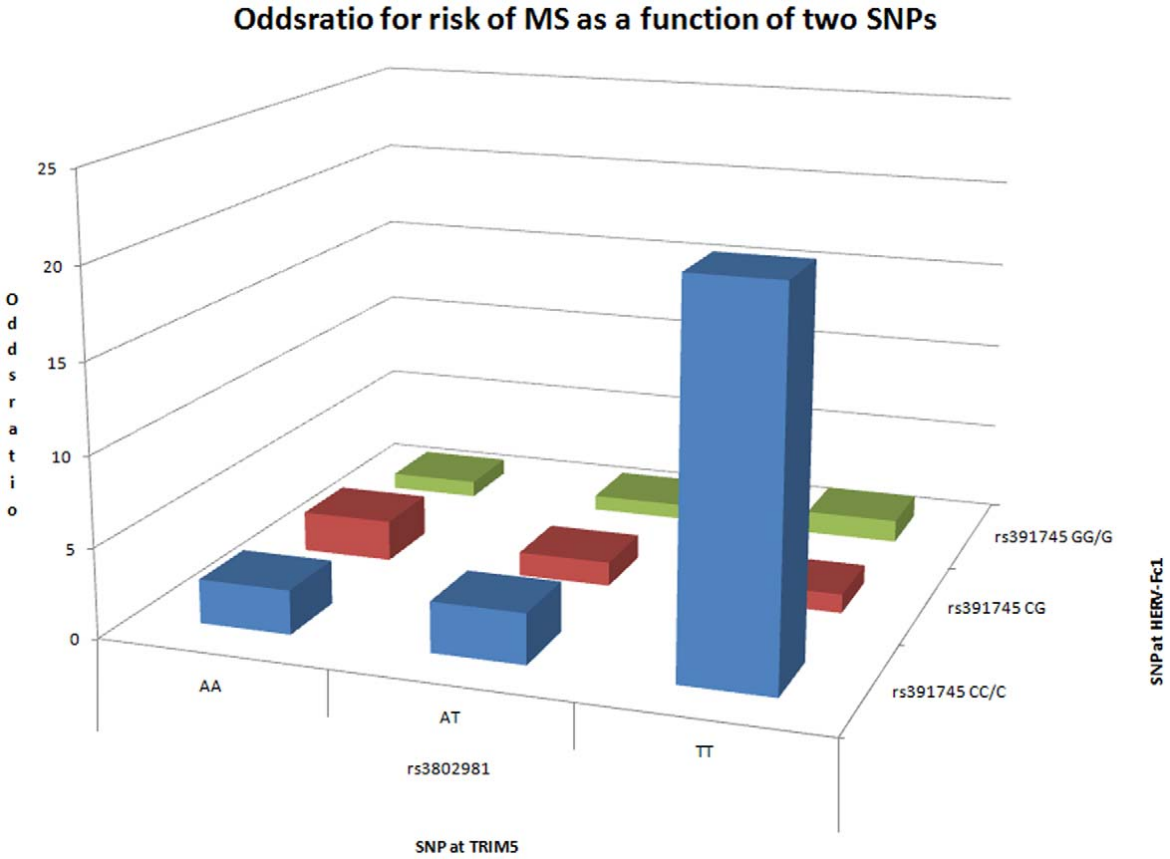
Odds ratio for MS in relation to 2 SNPs at HERV-Fc1 and *TRIM5*, respectively.

Looking further for an interaction or additive effect between HERV-Fc1 and *TRIM5* we depicted the odds ratio for disease as a function of the SNPs rs391745 and rs3802981, representing the two genes. The results are shown in Figure 2. It is obvious that disease risk is strongly influenced by the two genes, albeit largely in an additive fashion.

## Discussion

By treating the endogenous retroviruses as inheritable loci, we have brought the formidable knowledge of human genetics to bear on their role in disease, and avoided the difficulties of establishing the causality of a ubiquitous agent by conventional means. It is not clear if HERV-Fc1 is infectious, but analyses of the proviral DNA suggest the presence of two stop-codons and a frame-shift in the *pol* gene. The reading frames of the *gag* and *env* genes appear to be complete (NCBI build 37.1). We cannot exclude the contribution of other endogenous viruses to putative infectivity through pseudotyping or recombination. Perhaps the relevant determinant of infectivity in the organism is not the individual locus, but the repertoire of proviral sequences [13]. The p-values obtained are convincing arguments for an association of HERV-Fc1 with multiple sclerosis. However, the odds ratios are less striking than what could be hoped for. We attribute the latter to the fact that we have not yet found the causative variations, but depend on associations between close SNPs. The latter also applies to the results with *TRIM5*.

The gene *TRIM5* encodes an ubiquitin ligase that restricts the replication of many retroviruses to varying degrees [9]. The gene product accelerates unpacking of the viral cores and leads to abortive infections. In many ways, TRIM5 action resembles the effect of the mouse restriction gene *Fv-1*. The association of *TRIM5* with multiple sclerosis is independent confirmation that a retrovirus plays a role in the etiology of MS.

Our results should be seen in combination with previous findings in genomic scans that demonstrate that the major histocompatibility complex on chromosome 6 is important for this disease [14], as seems the receptor for interleukin 7 [15]. Retroviral infections often develop into running battles between the immune system and virus, with the virus mutating repeatedly to avoid the immune system, and the immune system repeatedly catching up [16]. One can see the episodic nature of multiple sclerosis as such a running battle. We also note that HERV-Fc1 is closely related to the HERV-H viruses previously described as activated in patients with multiple sclerosis [17].

Our suggestive evidence that a gene for multiple sclerosis is located on chromosome X could provide a natural explanation for the old observation that more women than men suffer from this disease. This is the normal pattern for X-dominant diseases. For Cohort 3 we obtained information about the clinical subtypes of the patients, but the cohort was too small for rigorous analyses of the subtypes. However, there were no signs of association with rs391745 among the patients with a primary progressive disease, and the association of the remaining (relapsing-remitting and secondarily progressing) was slightly stronger (p = 0.008) than that of the total cohort. This may indicate a different etiology of primary progressive sclerosis and is in accordance with the observation that the primary progressive form of multiple sclerosis has a sex ratio around 1.

In general, the finding that a disease is caused by an infectious agent is an encouraging one. These are the diseases, which we know best how to treat. If our results are confirmed, they point to new modes of treatment of Multiple Sclerosis.

## Materials and Methods

The studies were approved by the Science Ethical Committee of Mid-Jutland and were performed with written and oral consent from all persons. Four cohorts of ethnically Danish patients, with verified multiple sclerosis [9], were identified together with controls of similar ethnicity and gender from the same part of the country. A blood sample was drawn from each person and DNA was purified by conventional means. SNPs were tested using a Sequenom® platform (San Diego, California) and the iPLEX gold reaction. The SNPs, and the primers used, are listed in Table S1. Each PCR reaction contained 10 ng of genomic DNA, 0.5 U HotStart Taq from Qiagen (Hilden, Germany), 1.25×Enzyme Buffer (Qiagen), 3.5 mM MgCl_2_, and 1 mM of each deoxynucleotide. The primers were added to a final concentration of 500 nM each. The total reaction volume was 4 µl. PCR reactions with primers of each plex were performed in 384 well plates with V-shape by performing a 15 min preheat to 94 C followed by cycling the samples 45 times, 94 C for 20 sec, 56 C for 30 sec and 72 C for 1 min, followed by 3 min at 72 C. The plates were stored at −20 C. Treatment with Shrimp Alkaline Phosphatase and extension with molecular weight-modified nucleotides was performed according to the manufacturer's recommendations. The reactions were cleaned with resin and spotted on SpectroChip arrays according to the manufacturer's recommendations. The reactions were analyzed by MALDITOF mass spectrometry on the Sequenom equipment, and the results were analyzed using the MassARRAY Typer 4.0 (Sequenom). Significance was tested by a χ^2^ test (SPSS, IBM, Armonk, NY).

## Supporting Information

Figure S1 **A cartoon of the human genome with locations of the relevant endogenous viruses and indication of significant markers nearby.**
(TIF)

Table S1 SNPs used for the analysis of *TRIM5* and their primers.
(DOC)

Table S2 SNPs used for the analysis of *TRIM5* and their association with MS.
(DOC)

Table S3 SNPs used for the analysis of selected endogenous retroviruses and the primers used.
(DOC)

Table S4 SNPs used for the analysis of selected endogenous retroviruses and their association with MS.
(DOC)

Table S5 Association of the alleles of rs391745 with MS in the sex-stratified cohorts.
(DOC)
