# Supplementary figures and images for: The Etiology of Multiple Sclerosis: Genetic Evidence for the Involvement of the Human Endogenous Retrovirus HERV-Fc1
Source: PLoS One. 2011 Feb 2;6(2):e16652. doi: 10.1371/journal.pone.0016652 (PMC3032779; doi:10.1371/journal.pone.0016652)

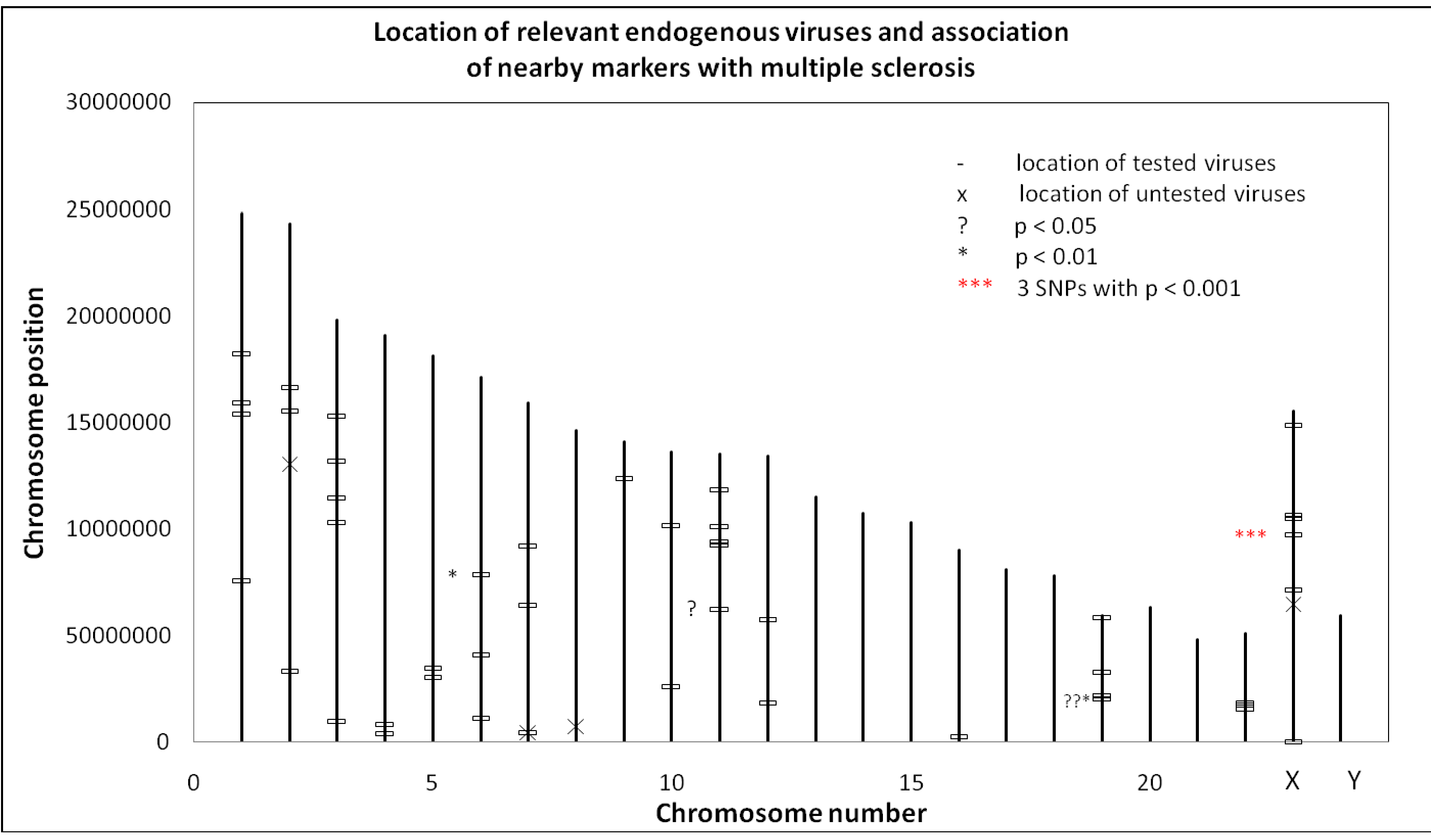

Supplement: Figure S1 — A cartoon of the human genome with locations of the relevant endogenous viruses and indication of significant markers nearby. (TIF) [file pone.0016652.s001.tif]
