# Supplementary material for: The Etiology of Multiple Sclerosis: Genetic Evidence for the Involvement of the Human Endogenous Retrovirus HERV-Fc1
Source: PLoS One. 2011 Feb 2;6(2):e16652. doi: 10.1371/journal.pone.0016652 (PMC3032779; doi:10.1371/journal.pone.0016652)
Supplement: Table S1 — SNPs used for the analysis of TRIM5 and their primers. (DOC) [file pone.0016652.s002.doc]

| SNP | PCR Primer 1 | PCR primer 2 | Extension primer |
| --- | --- | --- | --- |
| rs2133256 | ACGTTGGATGGAACTAGGGAAAGAACTCCA | ACGTTGGATGCCTTCATGGTGTGACAGC | GTGACAGCTGTCCAG |
| rs12287199 | ACGTTGGATGTTGACCCCTGACCAGATCCA | ACGTTGGATGAGGCAATTAGGGATTGTCAG | GGATTGTCAGTTCCAGT |
| rs7114084 | ACGTTGGATGTGACAAATGGAGATAACAGC | ACGTTGGATGATTCCTCTACTTCCACACCC | ccctACACCCCAATTGCTC |
| rs2880574 | ACGTTGGATGTGGAGCCTAAAGGTGTAGAC | ACGTTGGATGACATGTGTGGTCCACTGAG | TGGTCCACTGAGTATTTCT |
| rs1498553 | ACGTTGGATGCTTCCATAGATCTCATAGCC | ACGTTGGATGAGACCAGGATGAAAGTTCAC | gaAGATGTTCAAAGCCAGA |
| rs7124435 | ACGTTGGATGGAGCAAATGTGAAAGCCCTG | ACGTTGGATGTCTTTCCCACGCCAAAACAC | tACGCCAAAACACATCCTCA |
| rs12278842 | ACGTTGGATGCCAAAAGCAGAGCTTAGGTG | ACGTTGGATGTCCCAGCTACTTGGGTATTC | ccaccTGCTTCTCCTGCTCTT |
| rs7117107 | ACGTTGGATGCTGCTGATAATAAGAGCAATC | ACGTTGGATGGCTCTGACTACCTTATGTGC | GCAGTGTAACAAATCTCACCA |
| rs4992801 | ACGTTGGATGCCCAGTAAATCTGCATCTCG | ACGTTGGATGGATGATTCTGTTCCCGGA | cgagtGTCGGCTGCTGCTTCT |
| rs937446 | ACGTTGGATGTCCACCCTATAGGCTAACAC | ACGTTGGATGTCCCCCAAAACAGTGGAAAG | TTGGCTTTAAAAGAACTGAGTA |
| rs3802981 | ACGTTGGATGCGAAAAAGGGCACCTTTTCT | ACGTTGGATGATCATGCCACTTCTTCTGAC | TGCCACTTCTTCTGACATAGTTT |
| rs3802980 | ACGTTGGATGGAAATGCCATTGGCCCATAG | ACGTTGGATGGAGGAACCGCAGGAAATTC | tttctCAGGAAATTCTTGCTCAC |
|  |  |  |  |
