# Supplementary material for: The Etiology of Multiple Sclerosis: Genetic Evidence for the Involvement of the Human Endogenous Retrovirus HERV-Fc1
Source: PLoS One. 2011 Feb 2;6(2):e16652. doi: 10.1371/journal.pone.0016652 (PMC3032779; doi:10.1371/journal.pone.0016652)
Supplement: Table S2 — SNPs used for the analysis of TRIM5 and their association with MS. (DOC) [file pone.0016652.s003.doc]

|  |  |  |  |
| --- | --- | --- | --- |
| SNP | Chromosom | Position | P-value (Pearson, 2-sided) for association with MS |
| rs12278842 | 11 | 5701990 | 0.129 |
| rs7114084 | 11 | 5702343 | 0.086 |
| rs7117107 | 11 | 5702489 | 0.099 |
| rs4992801 | 11 | 5702489 | 0.099 |
| rs7124435 | 11 | 5703558 | 0.646 |
| rs12287199 | 11 | 5703870 | 0.288 |
| rs9374465 | 11 | 5705490 | 0.409 |
| rs2133256 | 11 | 5705675 | 0.091 |
| rs3802980 | 11 | 5706311 | 0.013 |
| rs3802981 | 11 | 5706523 | 0.004 |
| rs2880574 | 11 | 5709028 | 0.206 |
| rs1498553 | 11 | 5709819 | 0.005 |
