# Supplementary material for: The Etiology of Multiple Sclerosis: Genetic Evidence for the Involvement of the Human Endogenous Retrovirus HERV-Fc1
Source: PLoS One. 2011 Feb 2;6(2):e16652. doi: 10.1371/journal.pone.0016652 (PMC3032779; doi:10.1371/journal.pone.0016652)
Supplement: Table S3 — SNPs used for the analysis of selected endogenous retroviruses and the primers used. (DOC) [file pone.0016652.s004.doc]

| **MS1** | **primer 1** | **primer 2** | **extentionsprimer** |
| --- | --- | --- | --- |
| MS1_rs10266695 | ACG TTG GAT GTT CTT AGT GCC CCC TAT GAC | ACG TTG GAT GTG TTG CGG GGC TTA GAT ATG | CAG TTT GCG GGG CTT AGA TAT GAC ATA A |
| MS1_rs10985376 | ACG TTG GAT GGG TTC TTA TTT TTT TGC CTC C | ACG TTG GAT GGC ATT TGA GAG TAA CAC ACC | GTA ACA CAC CAC TAG TAT TAG |
| MS1_rs10985387 | ACG TTG GAT GAA TAA ATT ACA TCA AAG AAC | ACG TTG GAT GCA CGA CCC AGA GGA AAT TGC | AGG GTG ATG CAT CTG |
| MS1_rs1152326 | ACG TTG GAT GCT CAG ATC ACT GGC CCA TTT | ACG TTG GAT GAC TCA GAC TAT ACA AGG AAC | CCT CCA CAT CTC AAC AGC AAC AAA A |
| MS1_rs11523890 | ACG TTG GAT GCT CAC TTA TAC TAA TGC CAG | ACG TTG GAT GCA CCA TAC TTC TCT TCC TTG | CCT TGG TTT ACT GTA GGA |
| MS1_rs11754914 | ACG TTG GAT GGC TAA CCT ATT TCC CTA TGC | ACG TTG GAT GCC AGG AGG TGA CAA TAG TGC | TGG ACA CCA AAG GGG C |
| MS1_rs1249808 | ACG TTG GAT GGA CAA GAG ATC CCA TTA CCC | ACG TTG GAT GTC AAT CTC TGC CTC TGT CTG | CCC CGG CCT CTG TCT GGT CTC T |
| MS1_rs12611178 | ACG TTG GAT GGG GAT CAA CTA GAT CTT CAA C | ACG TTG GAT GGA GGA TTA CAA TTT TTT CTA G | ACT TAT AGT ACA TAA TTT AGG ATG AGG A |
| MS1_rs12823738 | ACG TTG GAT GGG GTA GAT GAT CTT TGC TGG | ACG TTG GAT GAT AAA AGT AAA GAA TAG AGC | AGT AAA GAA TAG AGC TTC ATC AG |
| MS1_rs170320 | ACG TTG GAT GCC ATT TAT AGC ACT GTA CAG C | ACG TTG GAT GTA GAC AGC TCT GCC CTC AAG | GCT TGC AAT ATT GGT AGT CTA TTA AAT |
| MS1_rs1822077 | ACG TTG GAT GTT CTT TGA TGA CTG GAT GTG | ACG TTG GAT GTG CAT GCG ACA ACG AGA CAG | CGG AAT GAG AAA ACA CCA CAG CAG G |
| MS1_rs1985254 | ACG TTG GAT GGA GAA GTA TGG TGG AAA GAT G | ACG TTG GAT GAA GCA TAA CCT CTT CCC CAC | ATT CCC CAC GTT AAA ATA GAA T |
| MS1_rs219073 | ACG TTG GAT GCC TCA AAG GCA AAG AGG AAC | ACG TTG GAT GTC ACA GTT CTG GAC TTC GAG | CGT CGA GGA TCC TTC TTC TGT ATT |
| MS1_rs2303473 | ACG TTG GAT GAC TGG AAA ACC TAG AGT GAC | ACG TTG GAT GTT CCA AGC TTA CTC AGC CTC | CTC AGC CTC TTG TAC TT |
| MS1_rs2435029 | ACG TTG GAT GCA TTG TGA CTT AGC ACT GGG | ACG TTG GAT GGT CAC AAG GTG CAC TGT AGG | CAC AAC CTC TCA TCC CCC AG |
| MS1_rs281040 | ACG TTG GAT GTG CCA CAT ATT ACT CCA TGC | ACG TTG GAT GGA GTC AGT CAC CAA GTT CAT | GAA CTT TAC ACA ATA TCC AAG GTC AA |
| MS1_rs2984350 | ACG TTG GAT GGA GCA ATA ATT ATC AAT GG | ACG TTG GAT GCT GTG AAT ATC CAG TTC CCC | ACC ACC AAC CCA TCA G |
| MS1_rs4618579 | ACG TTG GAT GAA GAG GCA TAA CTA TAG GAG | ACG TTG GAT GGT GGC TGG GCC ATA ATA TTG | CCC CTC AGG AGG CCA TTC A |
| MS1_rs4971095 | ACG TTG GAT GTG TCA GTG GCT GAT AGA AGG | ACG TTG GAT GGG AGT CTC TAC AAT CTG GAG | ACA ATC TGG AGT AAG TTT AGG |
| MS1_rs5920968 | ACG TTG GAT GGA CAG CGG GTC AAC ATT TAC | ACG TTG GAT GTC CTG CCA GAG AAC AGA ATG | AAA TAA TGC GTG TGT GCA ATG AGA AT |
| MS1_rs5962376 | ACG TTG GAT GTT GTT CAC CTA TGT GTC AAG | ACG TTG GAT GCT ATT CAC ATG GCT GCT AAA G | CAT GGA AAC AAC TGT AGT GCT TA |
| MS1_rs5993571 | ACG TTG GAT GAC AGT GGC CAA TAA TAC AGG | ACG TTG GAT GAT TCC TAA ACA CGG AGG AAG | ACA CGG AGG AAG TAA ATA G |
| MS1_rs603486 | ACG TTG GAT GGG GAG GAT ATG GTC ACT TTG | ACG TTG GAT GCC TGT GCA ACA TGT GGA TTC | CAT GTG GAT TCA GTG AGC |
| MS1_rs6460216 | ACG TTG GAT GTA AAC CAA AGC ACA ACT AC | ACG TTG GAT GGG CCG AAG TAC TTT TAT TGC | ATG TTT CAG TGT ATG AAC TGA ACA G |
| MS1_rs6619307 | ACG TTG GAT GTT TTC CAG TTG AGG AGG TCG | ACG TTG GAT GAG ATG AGG ATG GGC ACA TGG | TGT ACC AGC CCT TCT |
| MS1_rs6620400 | ACG TTG GAT GGG AGA GAT TAA CTA GGC CTG | ACG TTG GAT GAG GCA GGA CCG TTT TCA TGG | AGA ACC CTA GTC CGC CA |
| MS1_rs6712285 | ACG TTG GAT GCC CGC GCC AAG AAA ATA AAC | ACG TTG GAT GCC ATT GCA ATT AAG CAC TCC | CCT ATG AAC CAT TCC CGA AA |
| MS1_rs7933855 | ACG TTG GAT GGG AAA GAG AAC TGG GAT GAG | ACG TTG GAT GGA TTA GCC CAA ATC ATT CAG | AAA TCA TTC AGT CAA AGG CA |
| MS1_rs8110756 | ACG TTG GAT GCC ACC CCT GGA AGT CTA ATA | ACG TTG GAT GTG GGT TCG GAC AAA AGT CTC | GGA CAA GTC TCT CCC ATT AGA ATG C |
| MS1_rs9604952 | ACG TTG GAT GAC CTG GTC CGG CTT ACA GTT | ACG TTG GAT GCA GCC ACT TCT CTA AGA GGA | GAG GGG AAG CGC TAG TC |
| MS1_rs9823336 | ACG TTG GAT GAC CAC AAA AGA TCT TAA AC | ACG TTG GAT GAA TCT CTC TAA GCT TCA GCC | CTT TGC ATC TAT CAC ATA GAA AT |
| MS1_rs9840472 | ACG TTG GAT GAT CAC AGG AGA AAG GTG GAG | ACG TTG GAT GAT CAC CCC ACA ACA CCA CAG | CTT CTT GAG CCT TCA CTC TTC ATT |
|  |  |  |  |
| **MS2** | **primer 1** | **primer 2** | **extentionsprimer** |
| MS2_rs10416706 | ACG TTG GAT GTC CCC GAC CCA GGA ATT TTT | ACG TTG GAT GCA TAG CTG TGT AAC AAA CCC | CCA CAT GTG TAC CCC |
| MS2_rs10426414 | ACG TTG GAT GCA ATA CTC GCT ATG GCC AGA | ACG TTG GAT GTT AAC AGG CCA TGT CTA AGG | ATA GGC TCA AGT CCT TC |
| MS2_rs10818593 | ACG TTG GAT GAA CAG GCA CCA ACT GGT ATC | ACG TTG GAT GCT TTT AGG TAA AGT GGC CTG | TCC TGA ATA CAC AAT AAA TAT ACA AAA T |
| MS2_rs11172544 | ACG TTG GAT GAA GGG AAC CAC TGA TGC AAC | ACG TTG GAT GCA TCG AAA GAC TCA TGG CTG | GGC GCA AGA CTC ATG GCT GAC TTA CCT C |
| MS2_rs1174600 | ACG TTG GAT GGG TGT TCA CTG CTT AGG TAG | ACG TTG GAT GCA TGG AAA ACT CTC AGC ACC | CCA CAA CAT GCA TAC ACA TTG TC |
| MS2_rs11824690 | ACG TTG GAT GAA AAA GTT CCT AAA CAG G | ACG TTG GAT GCC AGG CAC CCA TAC CTA TG | GTT GAC GGA TAT TGC CGT A |
| MS2_rs12107596 | ACG TTG GAT GAA TTT ACG GCC TTA TGG GCT | ACG TTG GAT GCG GGC ATC TCG TAA CCA TAT | CTT GCC TCA TCG CAG |
| MS2_rs12117797 | ACG TTG GAT GAA GGT GGC GGG ATG AAT TTG | ACG TTG GAT GAT TGC CTG TAC CCA TTG TCC | CAT TGT CCT CCC CCA |
| MS2_rs12199776 | ACG TTG GAT GAC TCT CCT GGT CCT TTT TCC | ACG TTG GAT GTC ACA GCA ACC TTG AGA AAC | ATT GAG AAA CAG GAA TGT GC |
| MS2_rs12261856 | ACG TTG GAT GCT GTA CTG TGT CAT CAA GGC | ACG TTG GAT GAC AAT CAG ATA CCT AAA TG | CAG ATA CCT AAA TGT TGA TGT |
| MS2_rs16926345 | ACG TTG GAT GAC TTC TGA GTA CCC TCA GGC | ACG TTG GAT GGT GTG ATA AGT AGT AGC AAG | GGG CGT AGT AGC AAG TGG ACG |
| MS2_rs1939392 | ACG TTG GAT GGA GTA CAG TAT GAA AAC TGG | ACG TTG GAT GGC TGA CAC CGA ATA TTA ACC | GGG ACA TTG TAT AGT TAC TCT CCC |
| MS2_rs2091224 | ACG TTG GAT GAG CCT GCA TTT GCC TCC TTA | ACG TTG GAT GGC CAT CCC CAA TCA TAA GTC | TCA TAA GTC AAA TTA AGG GTA ATC T |
| MS2_rs2136918 | ACG TTG GAT GTT GAC CCT CAA ATG CTG AAG | ACG TTG GAT GCT CCT CTG GAA AGA GGA TAC | CTG TGG CCA GGT GGT |
| MS2_rs2182597 | ACG TTG GAT GAG AGG CCA CGT GGA TTT TTC | ACG TTG GAT GCA CCA GTC ACT GAT CTC ATC | CTA TCA ATT TCC CAG GAG CCG |
| MS2_rs2984351 | ACG TTG GAT GTG GAA GCA AGA GAG ATG AGG | ACG TTG GAT GTC CAT TGT ACT AGA TGC CTG | ATG GAC TAG ATG CCT GTT TCA T |
| MS2_rs352697 | ACG TTG GAT GGT CTG CTG GTA ACC ATG AAG | ACG TTG GAT GGT GAG ATT ATG GAA CTC CTG | ATG AGC TTC TTG GGT CAT TAA AG |
| MS2_rs3856908 | ACG TTG GAT GCA TCT TTG GGA GGT GAT GGG | ACG TTG GAT GGG CCT CTT GTA TAA GGG CG | CCC TTC TTG TAT AAG GGC GCT AAT CC |
| MS2_rs4827909 | ACG TTG GAT GTT GTC GTA TGC ATG TGA CCC | ACG TTG GAT GTT GTC CTT CCA TCA GAT GTC | GTG TTG GAG TTT CTT GG |
| MS2_rs5992338 | ACG TTG GAT GTT AGG GTC TCC CCG ACT AAG | ACG TTG GAT GAA AGG TAG TTA GAT CCC CCC | CCC CTG CTA GAT TGT TTC C |
| MS2_rs6518591 | ACG TTG GAT GTT GTA GTT TGG ATG GCT CCG | ACG TTG GAT GAG CCC AGG ACC ATA TCT ATG | GGC AAT CTT CCC TCT CCA AAC CTT T |
| MS2_rs6689503 | ACG TTG GAT GCT CTT TTC TAG GAC TAG CAG | ACG TTG GAT GGG CTT GGT ATA ACG GTT TGG | ACC GGT TTG GTG AGT AGA G |
| MS2_rs6787835 | ACG TTG GAT GAA CTG GGC AAA AAC TCT GGC | ACG TTG GAT GTG CAT GTA GTT TGG CCT TAG | GGG TTG CCT TAG GAA AAC AAG TCA TAG T |
| MS2_rs7524742 | ACG TTG GAT GCC AGG ACC TCT TTC TTT GTG | ACG TTG GAT GTT TAA TTG TGC TTA CCG AC | AAA CTT GTG CTT ACC GAC ATA GCA GTT |
| MS2_rs9468414 | ACG TTG GAT GAG GCA AGG ATC AGA CCA GAC | ACG TTG GAT GGT ATT TTG TCA TGG CAG CCC | ACG CCC TGG CAA ACT AAT AT |
|  |  |  |  |
| **MS3** | **primer 1** | **primer 2** | **extentionsprimer** |
| MS3_rs10214511 | ACG TTG GAT GTA TAC TCC TTT TCT TTC CCC | ACG TTG GAT GGG ACC TCT GTG CAA TTA GCC | TTT CAC TCA CTG TGC TGA C |
| MS3_rs10877101 | ACG TTG GAT GGT TGG AAG GTT TCC AAG CTC | ACG TTG GAT GAT GGG ACT GAG TAC AGT TAG | AAA AGT GAC GAA AGA GAA GA |
| MS3_rs11224962 | ACG TTG GAT GAT GAT GTG AGA GGG CCT ATG | ACG TTG GAT GTA GCA GAC CAC TCT CAA CTC | GGG AAT CCT AGA GCT GCC CT |
| MS3_rs11224968 | ACG TTG GAT GTA TAG CCC AGA CCC TGC TC | ACG TTG GAT GTG CAA TTT AGC TAT GCC CTG | CCC GCC CTG TCC CCA CAT |
| MS3_rs1148533 | ACG TTG GAT GGT GCT CCA TTT CTG ACA TTG | ACG TTG GAT GTC TCA GAG CAA CAG GGT ATG | CTG TAT CCT AAA CTC TTT AGA AAA TGA |
| MS3_rs12263936 | ACG TTG GAT GGG GCT AGG ACT GTA AAG TG | ACG TTG GAT GTA TCA CCC AAT CCA CTC AGG | GTG GCT GGC TCG TTT G |
| MS3_rs13073918 | ACG TTG GAT GGA AAT CAA GTC CCA GAG GTC | ACG TTG GAT GAG CTT GCT GCT CTG CTG TC | CCC ATG CTC TGC TGT CCT GAG TCC |
| MS3_rs1384625 | ACG TTG GAT GGA TTA TCA AGG CCA AGG GAG | ACG TTG GAT GAG GAA TGC TTA ACA CAC TGC | ACA CAC TGC TTC CTG |
| MS3_rs1650930 | ACG TTG GAT GTT GGA GAG TAT ATC CCT GCC | ACG TTG GAT GGA CCA ATT AAT CTT TTC CC | CTT GTC CCA TGA GTT CCT GAT A |
| MS3_rs167609 | ACG TTG GAT GAA TTG GCA GGT TAC ATG TTG | ACG TTG GAT GGA CAA ATA CAG ATC TAT TCC C | CAA ATA CAG ATC TAT TCC CAA ATA A |
| MS3_rs2435031 | ACG TTG GAT GAG CCT TCT ATC TGA ACT CCC | ACG TTG GAT GAC TGG GTC AAG CAA ATT GTC | TCA AAT TGT CAC ATT ACC CCT |
| MS3_rs281053 | ACG TTG GAT GCT CAG GTA TTC CTT TAT GAC | ACG TTG GAT GTA GGT AGA AGC ATG AGC TGG | GTT TTA GGT GGA TGC TTT G |
| MS3_rs3740073 | ACG TTG GAT GGG GAA GAA ACA AAT GGA GC | ACG TTG GAT GCA AGC ACG TGA ATA CAT ATC | GCT CCT GAT GCA CAG TTA TT |
| MS3_rs4148397 | ACG TTG GAT GAA GCC AGG GAG ATG GCA AG | ACG TTG GAT GAT GTT CCT TCC TCT GCA GAA | TCC TCT GCA GAA TAA TCC T |
| MS3_rs4805369 | ACG TTG GAT GCC CGA TGA CCA TAG GCA TTC | ACG TTG GAT GTC GCC CAG ATG TCT CTA TCC | TCT ACT TAG ACT GCC TTG GAT AG |
| MS3_rs4809040 | ACG TTG GAT GTA CCC TAC ATC CAA GAG CTG | ACG TTG GAT GAA GGA AAT CTG CAG GGT GGC | CCC CCA CCA CCA GTT TAA |
| MS3_rs530133 | ACG TTG GAT GAT TGA ACA GGA GTG GTC TGA | ACG TTG GAT GCC AAA TTT TGA GCT GCT GTG | GGG CGC TGC TGT GTT ATA GAG G |
| MS3_rs5748485 | ACG TTG GAT GCC AGT CCA CAG AAA TGC TTG | ACG TTG GAT GGT GAC CCA TAG TTA CCC CG | CCT CAT ACA CAG ACA ACT CAC CAA TCG T |
| MS3_rs5940190 | ACG TTG GAT GAA AAG TTC AGA TCT GCC GAC | ACG TTG GAT GTT TTT GAT CCC AGG CTG CAC | TGC ACT CCC CCT GCC |
| MS3_rs598645 | ACG TTG GAT GGA GTT TAC TCC TGA ATG ACC | ACG TTG GAT GAG CTC CAA GAA GAA AGA GCC | ACA AGA AGA AAG AGC CAT AAA CTT |
| MS3_rs6620397 | ACG TTG GAT GTA CCG TCA ATC CCC ACT ACC | ACG TTG GAT GGC TAC CTA TTC TGT TTT GCC | GTT TTG CCT TCC TTC GG |
| MS3_rs6791696 | ACG TTG GAT GTC CCA CTA CAC AGT TGT ACC | ACG TTG GAT GCT CAG CCC GAA AGG GTT AAT | GAA GGC TGG GTT CAA G |
| MS3_rs7253937 | ACG TTG GAT GGA AAC CTA CAC CTG TAC TGC | ACG TTG GAT GTT TTA AGG TGG GAG AGT AGC | TTA AGC CAC AGA GGA ACA |
| MS3_rs7254577 | ACG TTG GAT GCC CGT TAG AAT CCC TGA GAT | ACG TTG GAT GTC TAC TGG AAA ACA CTC CCG | CCC CTA AAA CAC TCC CGG CCA ACC |
| MS3_rs7650483 | ACG TTG GAT GTG GGG TTA TCA GAA TTT AG | ACG TTG GAT GGT GTT CTG TCC TCA GAG GTC | GTC TGG GTC CCA ATT C |
| MS3_rs8182505 | ACG TTG GAT GGC TTT TCC TGG GCT GCA TTT | ACG TTG GAT GGA GCT ACT CAC AGA ACT CAG | AAT CAG CAA AAC ACT GTA GTT A |
| MS3_rs9604911 | ACG TTG GAT GAC CAC TGC CAG TGC CAT ACT | ACG TTG GAT GAG ACA AAT CGA TAG AGA CAG | CGA GAG AGA CAG AAG TCA CAC |
|  |  |  |  |
| **MS4** | **primer 1** | **primer 2** | **extentionsprimer** |
| MS4_rs10232215 | ACG TTG GAT GGG CAT CAG TGA GCT GAA ATG | ACG TTG GAT GGG GTT TAA TCT GCT TCA GGC | CCT GCT TCA GGC AGA AAA AC |
| MS4_rs10792346 | ACG TTG GAT GCA GGA CCA GCA TGT GGT TAG | ACG TTG GAT GAG ACA GAT TCT GCC TGT ACC | GAG AGT CTG CCT GTA CCA AAT ATG |
| MS4_rs11092162 | ACG TTG GAT GAA ACT GCT GGG TTC TCG TC | ACG TTG GAT GCA GGC GGC TTC CAA TAA GAC | GGA ACT TTG ACA AAT TAC GGG A |
| MS4_rs12925045 | ACG TTG GAT GGG TTT TGT TTG TGT TTC CCC | ACG TTG GAT GAG TTA TGG TGC AAT TTT AA | CCC ACA CAG GCT GTA GT |
| MS4_rs1564653 | ACG TTG GAT GAT GTA TCC TAG GAC TAT GAG | ACG TTG GAT GGT CCC TGG TAA ATG CTA AAC | CTA TAT CTT CAA ATA ACT TAG CCT T |
| MS4_rs1651576 | ACG TTG GAT GCC CTG GGG AAA GGG AGA CC | ACG TTG GAT GGT GTC AAG AAA CAA CAC C | GGG TGA GCA GAC CGG GAA AG |
| MS4_rs210559 | ACG TTG GAT GTA GTG AAA GCT GGG TCC AGG | ACG TTG GAT GAA TGC TGG TAT ATT CAG TGC | CAT TCA TTG TTG GCA CCA C |
| MS4_rs2140336 | ACG TTG GAT GAA CTG TCT CCT GAA CAC TGG | ACG TTG GAT GAG TGA CAG ATC TGG AGG ACG | GTC CAG GAC CAG AGA |
| MS4_rs2504278 | ACG TTG GAT GCT TTC TCA GTG ATC TCG TGC | ACG TTG GAT GAG ACA AAT TCC CAG TTT CTC | CCC AGT TTC TCA TCT TTT TTC AAA AAT A |
| MS4_rs2652427 | ACG TTG GAT GTT CTC CTT CAC CCT GAG TGG | ACG TTG GAT GTC AGG GTA CAG AGA TAG GAG | CTC AAG AGA TAG GAG GTT GGG |
| MS4_rs2688228 | ACG TTG GAT GTG AGG ACG TGA TGT TTT CGG | ACG TTG GAT GAC ATG GAG TGA CCA CAT GAC | TCA TGA CTC AGC AAT TCC A |
| MS4_rs2902299 | ACG TTG GAT GGA TGA AGA TGC CAT GAA AAC | ACG TTG GAT GTA TAT GGG TAA GCC ACT GGG | GGG GCG CCA CTG GGA CTG GCT AAT TAT G |
| MS4_rs322119 | ACG TTG GAT GGC TGA ATT TCC CCC AAT ATC | ACG TTG GAT GAA AGG GAA TCG GGT TCC CTC | GAA CGT AGG AAA AAA GAA AGA CCA C |
| MS4_rs3872610 | ACG TTG GAT GGA TGG GCA GCT GAT GCC AA | ACG TTG GAT GCT AAC TTT GGT CAA ATC GAA | CCC TGT CTC CAA GCA CCA |
| MS4_rs4132355 | ACG TTG GAT GGG CTC AAA ACC AGA TGG AAG | ACG TTG GAT GGG GTC CAA GAT ATA GTT CAG | CAG ATA TAG TTC AGG CCA TTG |
| MS4_rs4517316 | ACG TTG GAT GAC TTC CTG TTT ACA GGA GAC | ACG TTG GAT GGA CGG GCT GAG GCT TAA AAG | TTC GCC AAA TGC GGA CAG GG |
| MS4_rs4679676 | ACG TTG GAT GGA GCT TAG CCT AGA GAA AAA C | ACG TTG GAT GCA TAC ACC CAG AAG TAT AGG | ATA GGG ATA ACA GAA GAC C |
| MS4_rs4679677 | ACG TTG GAT GGT TGG TCA TCT GGA TTA GTG | ACG TTG GAT GGC ACT GAC ATT CTG GAT CAC | CTC CTT CTG GAT CAC TTG CAG CCA CA |
| MS4_rs5983521 | ACG TTG GAT GAT GGG CAA CCT TAT CTA GAG | ACG TTG GAT GGT TTT GTC TGC GAC TCG TCC | CCG TCC TGC TAC ACC A |
| MS4_rs6451123 | ACG TTG GAT GTC AGT GCG CTC CGA GTG CT | ACG TTG GAT GAG TTG GAA AAT GCG TAC CCG | TAC CGG AGA CCT TAC CAT |
| MS4_rs6460219 | ACG TTG GAT GAA GAT CCC CTT CCT TTG ACC | ACG TTG GAT GGC CTT ATG TGT GTT ATG ACC | AGT CTT CAC CTG GGA |
| MS4_rs7650656 | ACG TTG GAT GTT GAC ACA CGC CCA GAC CTA | ACG TTG GAT GAT TGG GAC CCA GAC CTC TGA | CCC CCG AGG ACA GAA CAC CAT CC |
| MS4_rs819079 | ACG TTG GAT GCT TCA ACT GAT GTT CGT TTC | ACG TTG GAT GAC TCC AGC TTA GTC TGA AGG | GCA GGC TCA AGT GTT GC |
|  |  |  |  |
| **MS5** | **primer 1** | **primer 2** | **extentionsprimer** |
| MS5_rs10403221 | ACG TTG GAT GGT ATT GCA GGA GGC AGA TAG | ACG TTG GAT GTT CTG GAT CAG GAC CCC TTG | ACA GAA TCT CCT TAC CTG CT |
| MS5_rs11264397 | ACG TTG GAT GCA TCA GAG AAC TTG CCT AGC | ACG TTG GAT GTG GTG CTA CTA GGT TTC AAG | CCA CGT CAA GTT CCC TTC TCA TTG |
| MS5_rs11760888 | ACG TTG GAT GGT GGG GTA TGC CCC ACC AG | ACG TTG GAT GAG GAA GGA CAC CAC CCT ATG | GAC TGA GAG GCA GCC |
| MS5_rs12185748 | ACG TTG GAT GAC ATG CCT CCA TAG TTA ACC | ACG TTG GAT GGT AGG AAG CAG ACA AGA ACG | TGA TGA GAA CCA TGG TGT |
| MS5_rs12196881 | ACG TTG GAT GAG TCT CCA AAC ATT CCC TGC | ACG TTG GAT GTT TCC ATG AGG AGA AGC CAG | CGC CCA TAC TGT ACC ACT |
| MS5_rs1387153 | ACG TTG GAT GGT CTG TGG AAT GCT AGC AAA | ACG TTG GAT GGC TCA CAA CTT TGT GTT GCT | TGC TTT TAC AGA TAA GAA AAT TGA GTT |
| MS5_rs1651559 | ACG TTG GAT GTT GAT GGG TGC AGA GAT GTG | ACG TTG GAT GCA GCA CCC AGG TAA TGT AAC | GAC GGG GCC CTG TTT ACA TAA GAC |
| MS5_rs219200 | ACG TTG GAT GAC CAG GGT CAG TCT GGA TG | ACG TTG GAT GGT ACT TAT GTT TAC TTC TGT C | CTT ATG TTT ACT TCT GTC TAA AAA |
| MS5_rs2631731 | ACG TTG GAT GTG CCT CCA GTC TTC TCT GC | ACG TTG GAT GTC GGA GGA CTT TAC ACA TTG | CAC GAA AGG GAA GTG AT |
| MS5_rs2688242 | ACG TTG GAT GAA AGT GCC AGG CCC GGG G | ACG TTG GAT GCC TGC TCT GGG TAA CCC TG | CCC CGC CCG CGA ATC C |
| MS5_rs3088174 | ACG TTG GAT GTA TCC AAA CAG CCA TAG AGG | ACG TTG GAT GAG TTA GGG CCA TTA GTC GTG | AAT TAG TCG TGT GGT AGT C |
| MS5_rs4576878 | ACG TTG GAT GCT AGG CTG AAT ATG ATA GGG | ACG TTG GAT GCT GAT TCA TTT TTC TCT TGC | CTC TTG CTC AAT TTT CTA TAA AC |
| MS5_rs4686378 | ACG TTG GAT GTG GGC ACA GTG GTG CAT TTC | ACG TTG GAT GTC TTT TCC CCA CAA CCA AGG | GGG CAC CAA GGG CCC AGC TCC TAA CA |
| MS5_rs4717229 | ACG TTG GAT GTT CGT CAT CAA GTT CCA GGC | ACG TTG GAT GTA GAC TAC CTC CTA GCC CAG | TCC TAG GGA GTA TGC GGA AAG TTC A |
| MS5_rs4718180 | ACG TTG GAT GGT CAC AAA CCC TAT AGG TCC | ACG TTG GAT GGA ACT TGA GAG ATT TAT GG | GGG TTT GAG AGA TTT ATG GGA GTC C |
| MS5_rs4727276 | ACG TTG GAT GAG GAT ATA CCA CAA CGT CGG | ACG TTG GAT GGA GCC ACT TAT CCC CAC TTA | AAT CCG CCC ATT TCG ATT GTA ACA TCT |
| MS5_rs4764383 | ACG TTG GAT GAG TTC AAA TGG CAT GAT CAC | ACG TTG GAT GTT CCC TCT AGT ACA AGT GGC | TTG GCA GTC TAG GGT GAG AAA |
| MS5_rs5983522 | ACG TTG GAT GCT ATC AAA GTT TGG CAT G | ACG TTG GAT GGA GGA AGA TGA CTT CAT TC | CCT TTT GCC CAT AGA AAC CTT A |
| MS5_rs6501089 | ACG TTG GAT GGA CTG TTG AAT GCA TTT TTT | ACG TTG GAT GCC TGA TTT ACG CAG TAA AAG G | GCT TTG ACT CCT GAG GC |
| MS5_rs6620396 | ACG TTG GAT GCT CCA GGT AGC AGG TAA GTC | ACG TTG GAT GCC GAA GGA AGG CAA AAC AG | AAG TCA AAA CAG AAT AGG TAG CC |
| MS5_rs7586085 | ACG TTG GAT GCA AGG CAA CAA GCA TTG AAC | ACG TTG GAT GTC CAT TAA ATG CAC TTG CCC | GAC TTG CCC TCT GAT TGA A |
| MS5_rs9819214 | ACG TTG GAT GCC AGA ACT TCG GAA ATT GGC | ACG TTG GAT GGG GAA CAA CAG ACA GAA TAG | ACT CGC AGT CCA GAC |
|  |  |  |  |
| **MS6** | **primer 1** | **primer 2** | **extentionsprimer** |
| MS6_rs11797742 | ACG TTG GAT GTT CTC CTG GGT GAC TAC AAT | ACG TTG GAT GCT TGG GCT ACA GGA TTT ATT G | TTG GAT GTA GGG GAG A |
| MS6_rs11882251 | ACG TTG GAT GGG GTC AGC CAA GTG TTT TCA | ACG TTG GAT GAA TGG GAT AGC CTT TTA GC | ACT TTT AGC CAA CAC CAG |
| MS6_rs12013135 | ACGTTGGATGAGAGAACCACGGATGAACAG | ACGTTGGATGATATAAAAACATTTTGCTAC | cCATTTTGCTACTTAATAAAATTTAGA |
| MS6_rs12192983 | ACG TTG GAT GTC TGA CCT TCA CTA ACC CTG | ACG TTG GAT GGA TAC CTC CAC AAA AAC GGG | GAT CAG ATG TGA GTG GTG AT |
| MS6_rs1249822 | ACG TTG GAT GTT TGA AGT ATC TTG TCC ACC | ACG TTG GAT GAG GAA CTT AAC CAA ACA AG | TTA ACC AAA CAA GTA AAA AAC AA |
| MS6_rs16889290 | ACG TTG GAT GCA CTT TCC CAA ATC ACC CTC | ACG TTG GAT GGA GAA ATG AGG AAT TTT GCA G | AAT TTT GCA GGG GAG A |
| MS6_rs1929761 | ACG TTG GAT GAC AGT TTT CTT GGT GCT ATG | ACG TTG GAT GAA TTC AAC CCC AAC ACT GCC | CAA CAC TGC CTA TTA ATC AGA T |
| MS6_rs2096537 | ACG TTG GAT GGA AGC AAG ATC CTG ATG AAG | ACG TTG GAT GCT CCT GCA TAC AGG AAC TTC | GGA ACT TCA AAA TGC CTA AG |
| MS6_rs219077 | ACGTTGGATGGACTTTGCAAGAGGGAGACT | ACGTTGGATGGCTCAAAATTTCACTTCAGAG | AGTTAGTTTGAAAAGTCTACTATAA |
| MS6_rs219078 | ACG TTG GAT GGG GTA TTT GCA TTC AAG GT | ACG TTG GAT GGG GCT TAA ATT GTC CTC CAT | CAA GCC ACA TGT TAC TAA AAT C |
| MS6_rs2347214 | ACG TTG GAT GTC CAA GTT GCT TAC TCT CTC | ACG TTG GAT GAA TAG ACA AAC CCT CTA G | CAA ACC CTC TAG AGA CTA TA |
| MS6_rs2652425 | ACGTTGGATGGACACCTCTGAAACATGGGG | ACGTTGGATGCAGCCTTCCCTTGGTGTTTA | ttaagCATTGCAGGGATGCCGCT |
| MS6_rs4238842 | ACG TTG GAT GCA AAA GGT GTC AAC ACC CGC | ACG TTG GAT GGT CTT CTC TAA ACT CCC CCG | GAC CGC CCC CCT TTC |
| MS6_rs545075 | ACG TTG GAT GCA ACT ATT TGA AGG TAC TAC | ACG TTG GAT GTT GGT CAT TCA GGA GTA AAC | TGC CTG CCA GTC ACT |
| MS6_rs5748489 | ACG TTG GAT GTT AAA TGC AGT GCC TTC TCC | ACG TTG GAT GAG TGT GTG GAT GCC TGG AAC | ACA GAT GAC CGT TGC |
| MS6_rs5993426 | ACG TTG GAT GCA CCA CAT GCC CAA GTC TG | ACG TTG GAT GGT CTG TTC TCC AGC TCA GG | GGG CAG GAG ACA GGC AGA |
| MS6_rs7288876 | ACG TTG GAT GAG GCT TTG GTG TAT TGT TCC | ACG TTG GAT GCA AGC CCC ATA GGC TGA TTC | GGC TGA TTC AAG AAG ATA AA |
| MS6_rs9393931 | ACG TTG GAT GTG GCA AAA ACC ATA TCC AGC | ACG TTG GAT GAG ACT GCT CGC TAG TGG AAC | AGT GGA ACA CGA CAG |
|  |  |  |  |
| **MS7** | **primer 1** | **primer 2** | **extentionsprimer** |
| MS7_rs10126790 | ACG TTG GAT GCT ACT TAT TCT AAA GAA G | ACG TTG GAT GAC CGA ATC AAT CCT TCC TCC | CCC TAC CTC TGC CTG GAG AAA G |
| MS7_rs10426848 | ACG TTG GAT GGA ATG CAG GCA TTC TTT TGG | ACG TTG GAT GCC TAA GGT CAT CTA GAT GTC | CTA GAT GTC TCT ATA ATT AGA CTT T |
| MS7_rs1152324 | ACG TTG GAT GCA GTT TAG TCA GGC TTT TGG | ACG TTG GAT GCA TCT TTT GGC AAC TGG TGG | GGG GCC TTA AAG AGT TAT TTC AGT AAA C |
| MS7_rs1929772 | ACG TTG GAT GTG GAG GGA TGG GTG TAT AAG | ACG TTG GAT GAT GTG TGA ATA GGT GGC TGG | GTG GCT GGA GTC AAC AAA |
| MS7_rs2189362 | ACG TTG GAT GAG TGA TAG AGA AAC CTG GAC | ACG TTG GAT GCT CTA GGC TGC CTG GAA TTG | GAA TGA GTC TCT CCC AGT AGA AT |
| MS7_rs2379168 | ACG TTG GAT GGT GCT TAG TAG AAT GCT TTA | ACG TTG GAT GGG CAC AAG TAC ATG TTC AGT | AGT ATA AGC TAT AAT TAT TAT GAC GC |
| MS7_rs2396212 | ACG TTG GAT GGA AAG GTT TAA TTC TGT GAG | ACG TTG GAT GCT AGA ATT AAG CTA TCT GTG | GTA AGC TAT CTG TGA AAC TGC TC |
| MS7_rs318129 | ACG TTG GAT GGT AGA TGG AAG AAA CTC CAG | ACG TTG GAT GGA CTA AGG AAA CCA CTC TGC | ACC ACT CTG CTC TAG TT |
| MS7_rs318131 | ACG TTG GAT GGT GAG GAT CAC GGT GTA AGG | ACG TTG GAT GGG TGA TAG TGT CCT CCT TAA | CCT CCT TAA GGA ACT ACA G |
| MS7_rs318132 | ACG TTG GAT GGA GAG TAC ATC TGT CAA GCG | ACG TTG GAT GTA CCA AAG AGG CCC TCA AAC | CCC TGC CCT CAA ACT TAC TCT CA |
| MS7_rs318136 | ACG TTG GAT GTA TTG GAT CAA CGG GCA CAG | ACG TTG GAT GTC TCT TTG CTT CCT CTG CTG | GCA CTT AGC GAT GAC AC |
| MS7_rs318138 | ACG TTG GAT GCC TTA CTG AGG CCC TCA TC | ACG TTG GAT GGA CAT GAG TAG CCA AGA CAG | ATT AGG TGG GAT CAA GGC G |
| MS7_rs318156 | ACG TTG GAT GAA GGA GAA TTG GCT AGA AAG | ACG TTG GAT GTA TAG TGC CTG TCT TTC TGG | GTC TTT CTG GAC AGA GTT AG |
| MS7_rs318157 | ACG TTG GAT GCA GGA GGT TTT TCC TGA TTG | ACG TTG GAT GGT ACA GTC CAT GGA GTT CAG | CAC ATT CAC CCC CCG |
| MS7_rs391745 | ACG TTG GAT GGA TTC TCA GCA TGG ACC ATC | ACG TTG GAT GTA AAA TAT GTG CCC ACC CTC | GAC CTC ACA CTC AAC AGT ATC |
| MS7_rs400586 | ACG TTG GAT GGA TAT GTT TCC CAT GGA CCG | ACG TTG GAT GGG GTT TTA AGC CAG ACA GAA | GGG TGT AAG CCA GAC AGA AAT GGT A |
| MS7_rs445913 | ACG TTG GAT GGA ACC TCT GTC TGT AAT CGC | ACG TTG GAT GCG TCC TCG GTC CCG TAG TC | CCC ACT CGG TCC CGT AGT CGG AAA C |
| MS7_rs5964961 | ACG TTG GAT GGG TAT TCT TGT ACT TTA ACT G | ACG TTG GAT GGT TGA ATT TTC ACA AAA CAG G | TTA TTG AAG TTT AAT TCA TAT AAC ATA C |
| MS7_rs6615516 | ACG TTG GAT GCG CCA GTG TAC GTG CAA AAT | ACG TTG GAT GGA GAA GTG GTG GTC TCA TAC | TCC GAC CAT AGT GGA TTT GCC |
| MS7_rs6619304 | ACG TTG GAT GTG TCC CTT TGC TAC TAG CAC | ACG TTG GAT GAG ACA CTT GGT TAC ATC TGG | TCT CCA CTT GGT TAC ATC TGG TAG ACC |
| MS7_rs7116792 | ACG TTG GAT GCC ATC TCA AAA ATA AAT AAA AG | ACG TTG GAT GAC TCC TGA CCT CAA GTA ATC | ACC TCA AGT AAT CCA CCT G |
| MS7_rs7935912 | ACG TTG GAT GAG AGG TTT CCT TTG CAG TCC | ACG TTG GAT GAC AAC AGC CTG CCA ATT ACC | GGA ACA TGG CCT GTG AGG CTG |
|  |  |  |  |
|  |  |  |  |
| **MS8** | **primer 1** | **primer 2** | **extentionsprimer** |
| MS8_rs11085338 | ACG TTG GAT GAC ATC TCC TAT TGT GAG AGC | ACG TTG GAT GGG ATT AGG AGT AGA AGC CAC | AGT AGA AGC CAC ACT TAA A |
| MS8_rs12013135 | ACG TTG GAT GAG AGA ACC ACG GAT GAA CAG | ACG TTG GAT GAT ATA AAA ACA TTT TGC TAC | CAT TTT GCT ACT TAA TAA AAT TTA GA |
| MS8_rs318155 | ACG TTG GAT GGA GCT ACT CTT CTA CCA TCC | ACG TTG GAT GGA GAG TGA AGG AGA AGG TGG | GTG GTG TCC AAG ATG AT |
| MS8_rs5962377 | ACG TTG GAT GCC TAG AAA CAC ACA ACT ACC | ACG TTG GAT GCA ATC TCT TGT TAT AAG TC | TCT ATT CAG ATT TTT TCT TTG TG |
| MS8_rs6619299 | ACG TTG GAT GGA GAA GTG GTG GTC TCA TAC | ACG TTG GAT GCG CCA GTG TAC GTG CAA AAT | TTT ACG TGC AAA ATA AAG TCA |
| MS8_rs7109277 | ACG TTG GAT GCG CAG TTG AGT GTG GGT AAC | ACG TTG GAT GCC AGT TTA GGG TTA CAG TAG | GTT ACA GTA GCT CCC CT |
| MS8_rs7886111 | ACG TTG GAT GTT CAA GTC CTA TTA TTT AAG | ACG TTG GAT GCC AGG TTC ATC AGA TGA ATC | CTC TCT AGC AAC CAT AGC |
| MS8_rs9394742 | ACG TTG GAT GTT AAC CGT GTT GTT GGG GAC | ACG TTG GAT GAC AGT CCT TGC TCT TCC CAG | TGA AGT TCT GTT GTG GTT TA |
|  |  |  |  |
| **MS9** | **primer 1** | **primer 2** | **extentionsprimer** |
| MS9_env Fc(1)122 | ACG TTG GAT GTT GTC CTT CCA TCA GAT GTC | ACG TTG GAT GTT GTC GTA TGC ATG TGA CCC | TAC CCA GCT CCA TAC C |
| MS9_rs11092163 | ACG TTG GAT GTG GAT GAG GGC CTC AGT AAG | ACG TTG GAT GTT CAA GGG TCT GAC GAG AAC | GGA AGA CGA GAA CCC AGC AGT TT |
| MS9_rs11646366 | ACG TTG GAT GGC GCC TGA GAA TTC CAA AG | ACG TTG GAT GCT GTT CAA ACT GCT CAC TAT G | TTG AAG CAT GCC AGA AAA A |
| MS9_rs11672223 | ACG TTG GAT GGA GTC AAA ATC TCA ATG GTG G | ACG TTG GAT GCA CAC TCT GCA CAA AAA ACC | AAA CCT AGG CTC CAA TG |
| MS9_rs12845339 | ACG TTG GAT GGA GTG CTG GGA TCA AGC TG | ACG TTG GAT GTA CTG GCC TAC TAG GGC AAC | ATC AGG TGG TGG CTT A |
| MS9_rs16894097 | ACG TTG GAT GAC ACA GCC AGC CAG TAG TAG | ACG TTG GAT GGG GCT TGC CAT GCT TAT TTC | GGG GTT ATT TCT TGC ACT CTA GGA |
| MS9_rs16982583 | ACG TTG GAT GCT TTA AAA GTG TTC TGC GGG | ACG TTG GAT GCG GCA GTT GCT GTG TGT TTG | AAA CAG CAG ATG GTG GGA TT |
| MS9_rs16982595 | ACG TTG GAT GTG GTA TGA AAG GGT CAC TTG | ACG TTG GAT GTT TTA TAT GGC TTG CCA AC | CTT GCC AAC TGG TAC T |
| MS9_rs17003794 | ACG TTG GAT GTC AGA GAA GAC GCC GGA AG | ACG TTG GAT GCT AGT AGC TGT ACC CCT ACC | TTT CCC CCT ACC CCA GGC TTT T |
| MS9_rs17333695 | ACG TTG GAT GTT TCC CAC CGC TGG TAA TAG | ACG TTG GAT GTG TAC AAT AGC ACC TGT GCC | GTG CCC AGC CCG GAG |
| MS9_rs318130 | ACG TTG GAT GCC TTG TTA GGA CCA GTG AAC | ACG TTG GAT GGT AAC TAA TGG TCA GAG GCG | ACA TGC TTT ATT AAT GGA GCT CTC |
| MS9_rs318133 | ACG TTG GAT GCA ACT GGA GCT GTG AGT AAG | ACG TTG GAT GAC CAG CCC TCT GTC TGA TCC | GAG TGG CCC GCC ATT TCC |
| MS9_rs421531 | ACG TTG GAT GCC TGG GAT TGT GCT TTA TGG | ACG TTG GAT GGG CAA AGT ATT CCC CTT TTA G | CCC CTT TTA GCA CAT TAA TAA ATG AA |
| MS9_rs4786303 | ACG TTG GAT GCA AAC ACA GGC TGA TTC AAC | ACG TTG GAT GGC AAA ATC TTT GCA TAC AC | CCG ATC TTA AAT TCC CAG AA |
| MS9_rs4786309 | ACG TTG GAT GTT ACC AGG CGG ATC GTG G | ACG TTG GAT GAG TAG CGG GTG TTG ACA CCT | CCT TGA CAC CTT TTG CTA C |
| MS9_rs5920696 | ACG TTG GAT GAC TAT AAT ACT TTG GGA GGC | ACG TTG GAT GTC ACT GTG TTA ACC AGG ATG | CCC ATG ACC TTG TGA TCT GCC |
| MS9_rs628526 | ACG TTG GAT GGC TTG GAA TCT CCC ACC AGA | ACG TTG GAT GGG GAT AGG TTT CGA CTT CTC | GGA TGA AAG TGC CCG TCC |
|  |  |  |  |
| **MS10** | **primer 1** | **primer 2** | **extentionsprimer** |
| MS10_env fc(1)401 | ACG TTG GAT GTG TAC AAT AGC ACC TGT GCC | ACG TTG GAT GTT TCC CAC CGC TGG TAA TAG | TGA CCT CCT CAC TTG TTG |
| MS10_rs11646705 | ACG TTG GAT GGG GTG AAA TGC TAT CAT GTG | ACG TTG GAT GTT ACC CTC TCA CCT CCC CAA | CAC CTC CCC AAA TTT TT |
| MS10_rs1229931 | ACG TTG GAT GGA CAA ATC TTT CAG CCC ATC | ACG TTG GAT GAA AAA AAG AAA AAG CTC CG | AAA AAG CTC CGG TAA AAG |
| MS10_rs1628324 | ACG TTG GAT GCT CCT GCT TGC TCC TTT CCA | ACG TTG GAT GAG AGA GAA GAC TCA CAT CAA | CCA GAG ATA TGT CAC AAT GC |
| MS10_rs28546315 | ACG TTG GAT GAA CTA TGG AGG AGG GTG GTG | ACG TTG GAT GAG GCA AAT GAT TAG GAT GGG | GCT TAT GAA AGG GAT AAT CCA |
| MS10_rs318137 | ACG TTG GAT GAA TCA GCG GGA GAT ATT CGG | ACG TTG GAT GTA ACT AGG TCC TGT ATT GGG | TGA GGG CCT TCC TCC |
| MS10_rs440200 | ACG TTG GAT GTT TCC GAC TAC GGG ACC GAG | ACG TTG GAT GCC CAA CCT TCC TTG AAG CG | TTG AAG CGC GGA TGG C |
| MS10_rs4827910 | ACG TTG GAT GGT ATG CAT GTG ACC CAG CTC | ACG TTG GAT GGT AAG TTG TCC TTC CAT CAG | TCC CTT CCA TCA GAT GTC A |
| MS10_rs6615948 | ACG TTG GAT GAG GGC CTC TTT GGT AAG CTC | ACG TTG GAT GCA GCT TGA TCC CAG CAC TC | CAC TCG GGG CTG GCA |
| MS10_rs675270 | ACG TTG GAT GTC ATC TCA CAC TTA GAC TAC | ACG TTG GAT GTG GCG AAG AGG TGA TTT CAG | AAA GTT GAG ACT TAA GAT TAG AT |
|  |  |  |  |
| **MS11** | **primer 1** | **primer 2** | **extentionsprimer** |
| MS11_pos4208 | ACG TTG GAT GTT GCA CTA AAC GGT ACG CAC | ACG TTG GAT GTC CTT ATC CCG GTA AAC TCC | tcc aaG CCC ATT CTG CCC ATC |
| MS11_pos6464 | ACG TTG GAT GAT TTT TGA GAC GAA CGC GGG | ACG TTG GAT GCT GCT AGA ACA TAT CAT CCC | TTC CCC GAT CCC TGC |
| MS11_rs1548293 | ACG TTG GAT GCT CAT AAT CTT TCA AGA GGG | ACG TTG GAT GAT GTC CCT CAC TGA AGC AAC | cct aAC TGA AGC AAC AGC ATA C |
| MS11_rs2779642 | ACG TTG GAT GGT GAA ATT CCT TGA GGG CAG | ACG TTG GAT GAG AGC ATG TAA ATA TTT GG | gaA TGT AAA TAT TTG GAA TGC TCT |
| MS11_rs28546315 | ACG TTG GAT GAG GCA AAT GAT TAG GAT GGG | ACG TTG GAT GAA CTA TGG AGG AGG GTG GTG | tGC AAT CCC CTT TAA CCA C |
| MS11_rs36019431 | ACG TTG GAT GTG GTT TTG GAC AGC ACC ATC | ACG TTG GAT GTT TGC CCA GCC AGG CAA ATG | gct TGG GAG GTT TGA CTT T |
| MS11_rs4786304 | ACG TTG GAT GGG GAA CTG TTG AAT CAG CCT | ACG TTG GAT GAG GTG GGG CAG CAG TCA GTT | cca AGT CAG TTG CCA GCG |
| MS11_rs4827910 | ACG TTG GAT GGT ATG CAT GTG ACC CAG CTC | ACG TTG GAT GGT AAG TTG TCC TTC CAT CAG | CCT TCC ATC AGA TGT CA |
| MS11_rs4974767 | ACG TTG GAT GTT GAC AAA ACC TCC ATC GTC | ACG TTG GAT GTC TAC AGA GGT ATA TCC AGC | GCG ACT AGC GAG AAT TG |
| MS11_rs55802271 | ACG TTG GAT GGC ATT TCC CAG GTG TGT AAC | ACG TTG GAT GAG AAA CCA ACA GTC AGA CTA | ccc cGTC AGA CTA AAA TAG CCC AC |
| MS11_rs5920969 | ACG TTG GAT GAC CTT CTC CGC AAG CAC GC | ACG TTG GAT GTG GAG AGA GCA CAA CAG GG | tGG TAG GTT CGG GGA G |
| MS11_rs618561 | ACG TTG GAT GAA TGA ATT TTG GGG CTT GGG | ACG TTG GAT GCA GGA GAA TGA GAT GGA TTA | cca cCT TAT TAA CTA CTG CTA TGG TC |
| MS11_rs6419443 | ACG TTG GAT GTG AAA CCC TAG CCT GAA CAA | ACG TTG GAT GGC GCA GTC ATT AAG TCA ATA | cgg ttC CCG CTC GGG ATT TCT AAG TTC |
| MS11_rs6431123 | ACG TTG GAT GCT AAT GAC CTC AGA ACT TGG | ACG TTG GAT GTA GTT CTT TCA ACT ATG GC | CAA CTA TGG CCT GTC C |
| MS11_rs6709481 | ACG TTG GAT GTG AGG CCA ACA TTC ATG TTC | ACG TTG GAT GCT GAG ACA ATA AGC CAT AT | tAG ACA ATA AGC CAT ATA TTC TGA A |
| MS11_rs6817808 | ACG TTG GAT GCA TGA GGG GGA AAA GTA AAC | ACG TTG GAT GAG TGT AAA AGG CAG CTG TAG | caa cTT GTC AAT GAC CCT GT |
| MS11_rs71534260 | ACG TTG GAT GGT CAC CTA TGA TGA CAA GGC | ACG TTG GAT GAT ATT AAC AAG GCG AGC CCC | GAC TAG GGT TAT CCA TGA AAA |
| MS11_rs71539636 | ACG TTG GAT GAG CTC TTC CCA AGA TCT CTC | ACG TTG GAT GGT CTC TGT AGA GAG GCT AAG | aGG CTG AGG CTT CGA T |
| MS11_rs71539637 | ACG TTG GAT GTT ACT TCT CAT AGC CCC CTG | ACG TTG GAT GAT TTG GTT GAC CGT CAC CCG | ccc ctG TCA CCC GGG AGA CTT C |
| MS11_rs73260457 | ACG TTG GAT GTG TCT TCC TCT TTC ACC TCG | ACG TTG GAT GCC ATC TCT AAT GCC ACT TTT | ggg gTT GTT TGT TTG CCT AAG TGT TTC C |
|  |  |  |  |
| **MS12** | **primer 1** | **primer 2** | **extentionsprimer** |
| MS12_pos1223-T | ACG TTG GAT GGT AAT TGC CTC TAA ATG GTT | ACG TTG GAT GGA ATG AAA ACA AGA AAA AGG | caA AAA CAA GAA AAA GGA AAA GG |
| MS12_Pos8494 | ACG TTG GAT GAA TAA CAG CAC TCT TCC CCG | ACG TTG GAT GAG CCC TAG ATC TCC TCA CAG | CGA CAA AGG CGG AAC |
| MS12_rs12971429 | ACG TTG GAT GGG CAA ATA TAA AAT TTG CTT C | ACG TTG GAT GGT GCC ACC ATG TTC ACT TAC | TGT TCA CTT ACC ATA ACT CT |
| MS12_rs2125324 | ACG TTG GAT GAC ATG ACC ATG TGT TCA GGC | ACG TTG GAT GCA CTC AAG TAA ATT CTC TGC C | tcT TCT CTG CCT GAT ATT CA |
| MS12_rs318134 | ACG TTG GAT GTT TGC TTC CTC TGC TGG CAC | ACG TTG GAT GAC TAT TGG ATC AAC GGG CAC | CAA CGG GCA CAG GGA GC |
| MS12_rs318190 | ACG TTG GAT GCT CTA CCA TTG CAA TCC ATC | ACG TTG GAT GCG AAT ACC AAG TGT GGA GTG | tca atA TGA CAC CCA CAG ATC TA |
| MS12_rs387312 | ACG TTG GAT GAA TGA TTG TTA TTG CTG CT | ACG TTG GAT GGA CCA CAC ATT TTA TGT TCC | CCT AGT GGT AAA AAA TAA AAA TAA AAA |
| MS12_rs4266790 | ACG TTG GAT GGT GCC TCC ACT GCT AAA ATA | ACG TTG GAT GTC ATA AGA AAT GAA ACT AC | TAA GAA ATG AAA CTA CTT TAG ACA |
| MS12_rs4827909 | ACG TTG GAT GTT GTC GTA TGC ATG TGA CCC | ACG TTG GAT GTT GTC CTT CCA TCA GAT GTC | TGT GTT GGA GTT TCT TGG |
| MS12_rs55748203 | ACG TTG GAT GTG CTA GAC ACT GGG AAC ATC | ACG TTG GAT GAC CAG TTG TTG AAG GAA GTC | ggg acG GGG TGC AGG CCC CA |
| MS12_rs56753401 | ACG TTG GAT GGG CAT TAG AGA TGG TCT TCC | ACG TTG GAT GAG TGG TCA CAA GCC TTT ACG | CGG TAA TTT ATG CTT CCA T |
| MS12_rs5916942 | ACG TTG GAT GCT CTC TTT CCT CTT TAG GGC | ACG TTG GAT GTG AAG CAT ACC TGC AAG ATG | CAT ACC TGC AAG ATG TAG AAA A |
| MS12_rs6540338 | ACG TTG GAT GGG CTG AGG GAG ATA GAA GTG | ACG TTG GAT GTC CCG CTA TAC ACA GAA GAG | GGG AGG AGG AAC CAT |
| MS12_rs6752853 | ACG TTG GAT GCC CAC ACA AAT GTA ACT GAA C | ACG TTG GAT GGT AGT AAG TCA TTA TGT TGG | ATT ATG TTG GAT TAA GGG AAA TA |
| MS12_rs6781014 | ACG TTG GAT GCT TCT TTT ATC GGA CTT ACC | ACG TTG GAT GCA TGA CAT GGA ACC CTT CAA | ggT CAA AAA TGA TGA CCC AAAA |
| MS12_rs6907006 | ACG TTG GAT GCA GCA GAG GAG AAA GAC CAG | ACG TTG GAT GAA TAG TGA GGG CCA TCT AGC | CTA GTC CAG ACA GAC GA |
| MS12_rs73199783 | ACG TTG GAT GAT GGC AAG TAA AAC ACA GGG | ACG TTG GAT GAT GAG TTT GGA TCT CAG TGC | ccc GGA TCT CAG TGC TAA TCT TTA |
| MS12_rs73544488 | ACG TTG GAT GCT GAT GAC GAT GTT ATC TTG C | ACG TTG GAT GGA TCT GTA TTT GTC TGT TAA G | TGT TAA GAA AGG AAT TAA AAA AGA G |
| MS12_rs7891183 | ACG TTG GAT GAG GAC TCT GCA TTC CTT CTC | ACG TTG GAT GGA TAG AGA CCT TTC AGA CGG | ggg aCT TTC AGA CGG AGA ATT CAA A |
|  |  |  |  |
| **MS13** | **primer 1** | **primer 2** | **extentionsprimer** |
| MS13_rs10838534 | ACG TTG GAT GTA GAT ATG AAG ATA TAT AGC | ACG TTG GAT GTA CAG CAA GAA TAG AAC AG | CAG AAA TGT GAC ATC AAG ATA |
| MS13_rs12278842 | ACG TTG GAT GTC CCA GCT ACT TGG GTA TTC | ACG TTG GAT GCC AAA AGC AGA GCT TAG GTG | AAG CAG AGC TTA GGT GAG ATA C |
| MS13_rs12287199 | ACG TTG GAT GTT GAC CCC TGA CCA GAT CCA | ACG TTG GAT GAG GCA ATT AGG GAT TGT CAG | AGG GAT TGT CAG TTC CAG T |
| MS13_rs1332885 | ACG TTG GAT GGT CTT GTT TTC TGG TTT GGC | ACG TTG GAT GGA CTG AAG ACT TCT TGA TGA C | TTC ACT CAA GAT TAC TTT CAC T |
| MS13_rs1332886 | ACG TTG GAT GCC AAA CTG ACT TTG CTG AGG | ACG TTG GAT GCA GTG AAG TGG GTA AAG GAG | GGG GAT AGG AGA AAC TTG |
| MS13_rs17305868 | ACG TTG GAT GAA AAT CGT ACT AAC ATC AG | ACG TTG GAT GCC AAA CAG CTT ATG CCT CAG | tCT CTT CTC TCA GCA TGA A |
| MS13_rs2342386 | ACG TTG GAT GGC AAA CCA AAG ATA CAT GGA C | ACG TTG GAT GGT TTA AAG AAA GCT AAA ATA T | AAA GCT AAA ATA TAA TTT TTA AGT GTA G |
| MS13_rs2890093 | ACG TTG GAT GAC TTT TGC TGT ATT TCA TC | ACG TTG GAT GCA GGC TTT TAA GGT AAT AGA C | TAA GGT AAT AGA CTT CTG AGA TGA TA |
| MS13_rs318158 | ACG TTG GAT GAA GTC AGT TTG GTG ACT GGG | ACG TTG GAT GGA CTC TAG AAG AAA GAA GCC | CCA TTT TTC CAA AAT AAC ATT TAT T |
| MS13_rs318161 | ACG TTG GAT GAA AAG CTG CTT ATT AGG CAC | ACG TTG GAT GTA AAA GGC ATA ATG GGA AG | GGC ATA ATG GGA AGA AAT AA |
| MS13_rs318162 | ACG TTG GAT GGC CCT TCT TAC TTA AA GTG T | ACG TTG GAT GGA CTT GGG TCA TAA GCA AAC | aat cGT GTT CCT TCC ATT TGC AG |
| MS13_rs318166 | ACG TTG GAT GCC TTC TCT TAG GAA TTT GGA C | ACG TTG GAT GTC TCA GTT CAA CTG AAC AGC | ggt GCT GAG TCA CAA TCT CTG |
| MS13_rs318168 | ACG TTG GAT GGG AAA CTG CAT GTT AAT TG | ACG TTG GAT GAG ATG GTG CCT AGA ATC CTG | ATG CAG TGC CCT ACT T |
| MS13_rs318173 | ACG TTG GAT GAG ATG GAG CCA CCA TTA GCC | ACG TTG GAT GTC TGC TGA ACT CTC TGG GTA | aac gCT CTG GGT ATA TTC AGA AGC |
| MS13_rs36019431_- | ACG TTG GAT GTG GTT TTG GAC AGC ACC ATC | ACG TTG GAT GTT TGC CCA GCC AGG CAA ATG | CGT GGG AGG TTT GAC |
| MS13_rs3824949 | ACG TTG GAT GAA CAA GAG GAA CCT CAG CAG | ACG TTG GAT GTA CAT TAA CCA GGA TTC CAG | AGG ATT CCA GAA GCC ATA |
| MS13_rs5916943 | ACG TTG GAT GCT TCA TGT TTG CTC AAA ACA G | ACG TTG GAT GGC CCG GCC GTA ATA GTT TTT | CGT AAT AGT TTT TAA AAA TGG GTT |
| MS13_rs6419444 | ACG TTG GAT GGA AAT GGC ACA CTG GTT CTC | ACG TTG GAT GGT GAC CAC TTG TTA CCA CTG | ccc tTG TTG TTT GTC ACA TCC A |
| MS13_rs6431122 | ACG TTG GAT GCA CTT TTA AAC CCT TAG GAG | ACG TTG GAT GTA GTT CCT TCA CCG TGC TTC | GCT TCC AAC CAA ATT GC |
| MS13_rs7127767 | ACG TTG GAT GAG ACA TAA TAA GTG ATT TAC | ACG TTG GAT GGA AAG AGG ACC CTG AAT ACG | GGT CAT CTC AGG TCA CCA TA |
| MS13_rs9874803 | ACG TTG GAT GTC AAA CCC AAA CAA CCT GGC | ACG TTG GAT GGG GTG GTA TTT GTT AGG GTG | AGG GTG AAG GGC ATA |
|  |  |  |  |
| . |  |  |  |
|  |  |  |  |
|  |  |  |  |
|  |  |  |  |
| Extension primers | konc. in primer mix | final konc. in reaktion |  |
| 1 | 7µM | 0,625µM |  |
| 2 | 14µM | 1,25µM |  |
| 3 | 8µM | 0,84µM |  |
| 4 | 10µM | 1,04µM |  |
| 5 | 12µM | 1,25µM |  |
|  |  |  |  |
| Primer concentrations | 0,5µM | 0,1µM |  |
