# Supplementary material for: The Etiology of Multiple Sclerosis: Genetic Evidence for the Involvement of the Human Endogenous Retrovirus HERV-Fc1
Source: PLoS One. 2011 Feb 2;6(2):e16652. doi: 10.1371/journal.pone.0016652 (PMC3032779; doi:10.1371/journal.pone.0016652)
Supplement: Table S4 — SNPs used for the analysis of selected endogenous retroviruses and their association with MS. (DOC) [file pone.0016652.s005.doc]

| ORF | coodinates | SNP_RS# | Position | p-value |
| --- | --- | --- | --- | --- |
| GAG_42911 | chr1:75616199-75620806 | RS1249808 | 75611733 | 0,867 |
| GAG_42911 | chr1:75616199-75620806 | RS2347214 | 75613626 | 0,126 |
| GAG_42911 | chr1:75616199-75620806 | RS1249822 | 75630606 | 0,564 |
| POL_57916 | chr1:153864005-153869320 | RS4971095 | 153872848 | 0,703 |
| POL_57916 | chr1:153864005-153869320 | RS11264397 | 153878109 | 0,64 |
| POL_46513_46510 | chr1:158930168-158932972 | RS352697 | 158926985 | 0,405 |
| POL_46513_46510 | chr1:158930168-158932972 | RS530133 | 158937311 | 0,772 |
| ENV_46511 | chr1:158933585-158935421 | RS352697 | 158926985 | 0,405 |
| ENV_46511 | chr1:158933585-158935421 | RS530133 | 158937311 | 0,772 |
| GAG_59414_59411 | chr1:181942167-181943798 | RS4517316 | 181938675 | 0,711 |
| GAG_59414_59411 | chr1:181942167-181943798 | RS6689503 | 181940920 | 0,652 |
| GAG_59414_59411 | chr1:181942167-181943798 | RS12117797 | 181943414 | 0,66 |
| GAG_59414_59411 | chr1:181942167-181943798 | RS2182597 | 181944496 | 0,69 |
| GAG_75790_75789 | chr2:33133415-33134961 | RS219200 | 33127156 | 0,568 |
| GAG_75790_75789 | chr2:33133415-33134961 | RS219073 | 33132635 | 0,506 |
| GAG_75790_75789 | chr2:33133415-33134961 | RS219077 | 33138319 | 0,024 |
| GAG_75790_75789 | chr2:33133415-33134961 | RS219078 | 33138974 | 0,485 |
| ENV_80419 | chr2:130436925-130439130 | RS6752853 | 130435297 | 0,968 |
| ENV_80419 | chr2:130436925-130439130 | RS6431123 | 130439083 |  |
| ENV_70149 | chr2:155436977-155438642 | RS2652425 | 155432866 | 0,613 |
| ENV_70149 | chr2:155436977-155438642 | RS2652427 | 155433092 | 0,226 |
| ENV_70149 | chr2:155436977-155438642 | RS6712285 | 155439956 | 0,986 |
| POL_82112 | chr2:166274997-166277340 | RS12185748 | 166285202 | 0,607 |
| POL_82112 | chr2:166274997-166277340 | RS7586085 | 166285735 | 0,593 |
| GAG_82130_82114 | chr2:166278922-166280571 | RS12185748 | 166285202 | 0,607 |
| GAG_82130_82114 | chr2:166278922-166280571 | RS7586085 | 166285735 | 0,593 |
| GAG_97575 | chr3:9867706-9870019 | RS3856908 | 9863958 | 0,655 |
| GAG_97575 | chr3:9867706-9870019 | RS4686378 | 9871259 | 0,759 |
| GAG_97575 | chr3:9867706-9870019 | RS13073918 | 9873612 | 0,993 |
| GAG_90991 | chr3:102894274-102896539 | RS2303473 | 102887503 | 0,221 |
| GAG_90991 | chr3:102894274-102896539 | RS6791696 | 102891786 | 0,235 |
| POL_90990 | chr3:102898243-102900598 | RS6791696 | 102891786 | 0,235 |
| ENV_103887_103885 | chr3:114226730-114231276 | RS9874803 | 114220477 | 0,231 |
| ENV_103887_103885 | chr3:114226730-114231276 | RS3872610 | 114222835 | 0,969 |
| ENV_103887_103885 | chr3:114226730-114231276 | RS7650656 | 114237277 | 0,423 |
| ENV_103887_103885 | chr3:114226730-114231276 | RS7650483 | 114237316 | 0,488 |
| ENV_103887_103885 | chr3:114226730-114231276 | RS6781014 | 114239776 | 0,83 |
| GAG_103883 | chr3:114231787-114234139 | RS3872610 | 114222835 | 0,969 |
| GAG_103883 | chr3:114231787-114234139 | RS7650656 | 114237277 | 0,423 |
| GAG_103883 | chr3:114231787-114234139 | RS7650483 | 114237316 | 0,488 |
| GAG_103883 | chr3:114231787-114234139 | RS6781014 | 114239776 | 0,83 |
| POL_104696_104695 | chr3:131648299-131650683 | RS9840472 | 131643559 | 0,421 |
| POL_104696_104695 | chr3:131648299-131650683 | RS322119 | 131653827 | 0,191 |
| GAG_104694 | chr3:131650757-131652707 | RS9840472 | 131643559 | 0,421 |
| GAG_104694 | chr3:131650757-131652707 | RS322119 | 131653827 | 0,191 |
| GAG_104694 | chr3:131650757-131652707 | RS819079 | 131660756 | 0,104 |
| GAG_94224_94222 | chr3:153006933-153008645 | RS4679676 | 153003634 | 0,681 |
| GAG_94224_94222 | chr3:153006933-153008645 | RS12107596 | 153008014 | 0,802 |
| GAG_94224_94222 | chr3:153006933-153008645 | RS4679677 | 153011371 | 0,285 |
| ENV_107739 | chr3:168023718-168025407 | RS6787835 | 168021018 | 0,937 |
| ENV_107739 | chr3:168023718-168025407 | RS9823336 | 168031119 | 0,719 |
| POL_125391_125393 | chr4:4033237-4035374 | RS6817808 | 4027305 | 0,067 |
| POL_125391_125393 | chr4:4033237-4035374 | RS4974767 | 4031034 | 0,17 |
| POL_109894_109886 | chr4:8475790-8477937 | RS2688228 | 8472245 | 0,511 |
| POL_109894_109886 | chr4:8475790-8477937 | RS2140336 | 8475731 | 0,189 |
| POL_109894_109886 | chr4:8475790-8477937 | RS2631731 | 8480717 | 0,458 |
| POL_109894_109886 | chr4:8475790-8477937 | RS2688242 | 8480843 | 0,681 |
| POL_153312_153315 | chr5:30526649-30529036 | RS4132355 | 30517045 | 0,976 |
| ENV_153616_153615 | chr5:34497638-34499650 | RS1822077 | 34500112 | 0,754 |
| ENV_153616_153615 | chr5:34497638-34499650 | RS6451123 | 34503431 | 0,763 |
| POL_160232 | chr5:156019554-156025633 | RS281040 | 156013327 | 0,208 |
| POL_160232 | chr5:156019554-156025633 | RS281053 | 156031287 | 0,231 |
| ENV_171089 | chr6:11211915-11213535 | RS9468414 | 11204827 | 0,129 |
| ENV_171089 | chr6:11211915-11213535 | RS9393931 | 11211874 | 0,748 |
| ENV_171089 | chr6:11211915-11213535 | RS11754914 | 11214985 | 0,757 |
| ENV_171089 | chr6:11211915-11213535 | RS10214511 | 11215201 | 0,225 |
| ENV_172316_172311 | chr6:40937843-40939438 | RS6907006 | 40929002 | 0,376 |
| ENV_172316_172311 | chr6:40937843-40939438 | RS1548293 | 40929435 | 0,051 |
| ENV_172316_172311 | chr6:40937843-40939438 | RS1929772 | 40931842 | 0,003 |
| ENV_172316_172311 | chr6:40937843-40939438 | RS16894097 | 40933078 | 0,029 |
| ENV_172316_172311 | chr6:40937843-40939438 | RS9394742 | 40935765 | 0,02 |
| ENV_172316_172311 | chr6:40937843-40939438 | RS12196881 | 40936923 | 0,026 |
| ENV_172316_172311 | chr6:40937843-40939438 | RS12199776 | 40947244 | 0,797 |
| ENV_172316_172311 | chr6:40937843-40939438 | RS1929761 | 40949306 | 0,271 |
| ENV_174695_174701 | chr6:78425058-78426806 | RS2504278 | 78423561 | 0,065 |
| ENV_174695_174701 | chr6:78425058-78426806 | RS16889290 | 78434669 | 0,397 |
| ENV_174741 | chr6:78484297-78488403 | RS1384625 | 78479707 | 0,446 |
| ENV_174741 | chr6:78484297-78488403 | RS12192983 | 78493573 | 0,397 |
| ENV_174741 | chr6:78484297-78488403 | RS1564653 | 78493843 | 0,268 |
| ENV_188274 | chr7:4598011-4603501 | RS11760888 | 4610919 | 0,336 |
| ENV_191393 | chr7:64089027-64090872 | RS6460216 | 64083079 | 0,906 |
| ENV_191393 | chr7:64089027-64090872 | RS3088174 | 64088861 | 0,685 |
| ENV_191393 | chr7:64089027-64090872 | RS4717229 | 64089134 | 0,495 |
| ENV_191393 | chr7:64089027-64090872 | RS4618579 | 64089398 | 0,487 |
| ENV_191393 | chr7:64089027-64090872 | RS6460219 | 64090571 | 0,506 |
| ENV_191393 | chr7:64089027-64090872 | RS2091224 | 64091457 | 0,924 |
| ENV_191393 | chr7:64089027-64090872 | RS4718180 | 64099761 | 0,795 |
| ENV_192333 | chr7:91936017-91937637 | RS10232215 | 91935102 | 0,366 |
| ENV_192333 | chr7:91936017-91937637 | RS10266695 | 91936712 | 0,408 |
| ENV_192333 | chr7:91936017-91937637 | RS4727276 | 91945688 | 0,535 |
| HERV K-115 | chr8q23.1:7364810-7365158 | rs73199783 | 7352334 | 0,399 |
| ENV_217394_217399 | chr9:123596678-123598254 | RS10985376 | 123590058 | 0,531 |
| ENV_217394_217399 | chr9:123596678-123598254 | RS10985387 | 123599611 | 0,352 |
| ENV_217394_217399 | chr9:123596678-123598254 | RS10818593 | 123600674 | 0,854 |
| GAG_229882_229881 | chr10:26101961-26103528 | RS12263936 | 26101450 | 0,943 |
| GAG_229882_229881 | chr10:26101961-26103528 | RS16926345 | 26104083 | 0,322 |
| GAG_229882_229881 | chr10:26101961-26103528 | RS12261856 | 26106596 | 0,353 |
| POL_233963_233964 | chr10:101573152-101577803 | RS2902299 | 101565250 | 0,623 |
| POL_233963_233964 | chr10:101573152-101577803 | RS3740073 | 101566950 | 0,506 |
| POL_233963_233964 | chr10:101573152-101577803 | RS4148397 | 101582310 | 0,637 |
| TRIM5 |  | rs12287199 | #REFERENCE! | 0,003 |
| TRIM5 |  | rs12278842 | #REFERENCE! | 0,004 |
| TRIM5 |  | rs17305868 | #REFERENCE! | 0,01 |
| TRIM5 |  | rs3824949 | #REFERENCE! | 0,032 |
| POL_245662_245655 | chr11:61900240-61902971 | RS11824690 | 61896043 | 0,439 |
| POL_245662_245655 | chr11:61900240-61902971 | RS7935912 | 61896448 | 0,372 |
| POL_245662_245655 | chr11:61900240-61902971 | RS10792346 | 61896645 | 0,037 |
| POL_245662_245655 | chr11:61900240-61902971 | RS7109277 | 61910322 | 0,594 |
| POL_246777_246773 | chr11:92319031-92321162 | RS1387153 | 92313476 | 0,837 |
| POL_246777_246773 | chr11:92319031-92321162 | RS1985254 | 92319361 | 0,639 |
| POL_246777_246773 | chr11:92319031-92321162 | RS11523890 | 92319426 | 0,955 |
| POL_246777_246773 | chr11:92319031-92321162 | RS7933855 | 92323970 | 0,718 |
| GAG_240359_240360 | chr11:93718839-93720500 | RS545075 | 93711326 | 0,095 |
| GAG_240359_240360 | chr11:93718839-93720500 | RS598645 | 93711393 | 0,872 |
| GAG_240359_240360 | chr11:93718839-93720500 | RS1939392 | 93729727 | 0,584 |
| POL_240934_240931 | chr11:101073916-101079430 | RS11224962 | 101067540 | 0,134 |
| POL_240934_240931 | chr11:101073916-101079430 | RS11224968 | 101069828 | 0,151 |
| ENV_248410_248405 | chr11:118097857-118099766 | RS603486 | 118092557 | 0,803 |
| ENV_250353_250355 | chr12:18245510-18247075 | RS4764383 | 18237964 | 0,469 |
| ENV_250353_250355 | chr12:18245510-18247075 | RS12823738 | 18245549 | 0,421 |
| ENV_250353_250355 | chr12:18245510-18247075 | RS4576878 | 18254172 | 0,126 |
| ENV_260042 | chr12:57008433-57010536 | RS1148533 | 57003705 | 0,66 |
| ENV_260042 | chr12:57008433-57010536 | RS11172544 | 57006996 | 0,603 |
| ENV_260042 | chr12:57008433-57010536 | RS1729803 | 57017115 |  |
| ENV_260042 | chr12:57008433-57010536 | RS10877101 | 57017164 | 0,357 |
| GAG_260041 | chr12:57013856-57016124 | RS11172544 | 57006996 | 0,603 |
| GAG_260041 | chr12:57013856-57016124 | RS1729803 | 57017115 |  |
| GAG_260041 | chr12:57013856-57016124 | RS10877101 | 57017164 | 0,357 |
| GAG_260041 | chr12:57013856-57016124 | RS1174600 | 57021979 | 0,431 |
| POL_294399_294401 | chr16:2653266-2655817 | RS11646705 | 2646557 | 0,089 |
| POL_294399_294401 | chr16:2653266-2655817 | RS4786303 | 2647450 | 0,144 |
| POL_294399_294401 | chr16:2653266-2655817 | RS4786304 | 2647483 | 0,078 |
| POL_294399_294401 | chr16:2653266-2655817 | RS4786305 | 2647518 | 0,04 |
| POL_294399_294401 | chr16:2653266-2655817 | RS4238842 | 2650849 | 0,041 |
| POL_294399_294401 | chr16:2653266-2655817 | RS11646366 | 2651823 | 0,674 |
| POL_294399_294401 | chr16:2653266-2655817 | RS12925045 | 2653403 | 0,05 |
| POL_294399_294401 | chr16:2653266-2655817 | RS6501089 | 2654987 | 0,018 |
| POL_294399_294401 | chr16:2653266-2655817 | RS12934809 | 2657793 | 0,007 |
| ENV_310016 | chr19:20341093-20343118 | RS4809040 | 20335506 | 0,959 |
| ENV_310016 | chr19:20341093-20343118 | RS7253937 | 20335655 | 0,236 |
| ENV_310016 | chr19:20341093-20343118 | RS12611178 | 20341764 | 0,021 |
| ENV_310016 | chr19:20341093-20343118 | RS11085338 | 20342210 | 0,174 |
| ENV_310016 | chr19:20341093-20343118 | RS8182505 | 20348930 | 0,414 |
| ENV_310016 | chr19:20341093-20343118 | RS10416706 | 20349698 | 0,358 |
| HERV K-113 | chr19q12:21841554-21841785 | rs12971429 | 21635186 | 0,22 |
| POL_314064_314063 | chr19:21857886-21860536 | RS2435031 | 21853448 | 0,229 |
| POL_314064_314063 | chr19:21857886-21860536 | RS2435029 | 21853998 | 0,013 |
| POL_314064_314063 | chr19:21857886-21860536 | RS1651559 | 21866025 | 0,65 |
| POL_314064_314063 | chr19:21857886-21860536 | RS1628324 | 21866377 | 0,775 |
| POL_314064_314063 | chr19:21857886-21860536 | RS11672223 | 21867620 | 0,467 |
| POL_314064_314063 | chr19:21857886-21860536 | RS1229931 | 21867990 | 0,07 |
| GAG_314062 | chr19:21861311-21864125 | RS2435031 | 21853448 | 0,229 |
| GAG_314062 | chr19:21861311-21864125 | RS2435029 | 21853998 | 0,013 |
| GAG_314062 | chr19:21861311-21864125 | RS1651559 | 21866025 | 0,65 |
| GAG_314062 | chr19:21861311-21864125 | RS1628324 | 21866377 | 0,775 |
| GAG_314062 | chr19:21861311-21864125 | RS11672223 | 21867620 | 0,467 |
| GAG_314062 | chr19:21861311-21864125 | RS1229931 | 21867990 | 0,07 |
| ENV_314652 | chr19:32821289-32823395 | RS2396212 | 32813469 | 0,006 |
| ENV_314652 | chr19:32821289-32823395 | RS11882251 | 32819041 | 0,006 |
| ENV_314652 | chr19:32821289-32823395 | RS8110756 | 32830238 | 0,091 |
| ENV_314652 | chr19:32821289-32823395 | RS4805369 | 32831216 | 0,1 |
| ENV_314652 | chr19:32821289-32823395 | RS7254577 | 32833006 | 0,015 |
| POL_314653_314655 | chr19:32824583-32826928 | RS11882251 | 32819041 | 0,006 |
| POL_314653_314655 | chr19:32824583-32826928 | RS8110756 | 32830238 | 0,091 |
| POL_314653_314655 | chr19:32824583-32826928 | RS4805369 | 32831216 | 0,1 |
| POL_314653_314655 | chr19:32824583-32826928 | RS7254577 | 32833006 | 0,015 |
| POL_314653_314655 | chr19:32824583-32826928 | RS2189362 | 32834087 | 0,016 |
| POL_314653_314655 | chr19:32824583-32826928 | RS10426848 | 32836340 | 0,087 |
| GAG_314651 | chr19:32827060-32829283 | RS11882251 | 32819041 | 0,006 |
| GAG_314651 | chr19:32827060-32829283 | RS8110756 | 32830238 | 0,91 |
| GAG_314651 | chr19:32827060-32829283 | RS4805369 | 32831216 | 0,1 |
| GAG_314651 | chr19:32827060-32829283 | RS7254577 | 32833006 | 0,015 |
| GAG_314651 | chr19:32827060-32829283 | RS2189362 | 32834087 | 0,016 |
| GAG_314651 | chr19:32827060-32829283 | RS10426848 | 32836340 | 0,087 |
| GAG_312152 | chr19:58204294-58205860 | RS10403221 | 58201519 | 0,243 |
| GAG_312152 | chr19:58204294-58205860 | RS1650930 | 58206139 | 0,243 |
| GAG_312152 | chr19:58204294-58205860 | RS10426414 | 58206416 | 0,261 |
| GAG_323159_323164 | chr22:15473027-15474549 | RS5993571 | 15469569 | 0,6 |
| GAG_323159_323164 | chr22:15473027-15474549 | RS7288876 | 15469639 | 0,196 |
| GAG_323159_323164 | chr22:15473027-15474549 | RS9604952 | 15473947 | 0,839 |
| GAG_323159_323164 | chr22:15473027-15474549 | RS2096537 | 15474749 | 0,971 |
| GAG_323254 | chr22:17307027-17309295 | RS9604911 | 17304956 | 0,796 |
| GAG_323254 | chr22:17307027-17309295 | RS5993426 | 17315609 | 0,914 |
| GAG_323254 | chr22:17307027-17309295 | RS5992338 | 17316399 | 0,219 |
| POL_323256_323253 | chr22:17310412-17314440 | RS9604911 | 17304956 | 0,796 |
| POL_323256_323253 | chr22:17310412-17314440 | RS5993426 | 17315609 | 0,914 |
| POL_323256_323253 | chr22:17310412-17314440 | RS5992338 | 17316399 | 0,219 |
| GAG_323272_323271 | chr22:18303511-18305346 | RS5748485 | 18303415 | 0,605 |
| GAG_323272_323271 | chr22:18303511-18305346 | RS6518591 | 18304021 | 0,24 |
| GAG_323272_323271 | chr22:18303511-18305346 | RS5748489 | 18307146 | 0,951 |
| POL_4490_4492 | chrX:64499269-64501515 | RS7886111 | 64495952 | 0,31 |
| POL_4490_4492 | chrX:64499269-64501515 | RS5964961 | 64496916 | 0,301 |
| POL_4490_4492 | chrX:64499269-64501515 | RS12013135 | 64505049 | 0,306 |
| ENV_4769 | chrX:71364907-71366560 | RS2984350 | 71369559 | 0,879 |
| ENV_4769 | chrX:71364907-71366560 | RS2984351 | 71371454 | 0,928 |
| GAG_8440_8437 | chrX:92572360-92573951 | RS6619299 | 92571294 | 0,766 |
| GAG_8440_8437 | chrX:92572360-92573951 | RS6615516 | 92571311 | 0,728 |
| GAG_8440_8437 | chrX:92572360-92573951 | RS6619304 | 92572635 | 0,75 |
| GAG_8440_8437 | chrX:92572360-92573951 | RS6619307 | 92573053 | 0,697 |
| GAG_8440_8437 | chrX:92572360-92573951 | RS5940190 | 92575134 | 0,853 |
| GAG_8440_8437 | chrX:92572360-92573951 | RS5983521 | 92580150 | 0,908 |
| GAG_8440_8437 | chrX:92572360-92573951 | RS5983522 | 92580206 | 0,974 |
| ENV_8436_8443 | chrX:92578106-92579623 | RS6619299 | 92571294 | 0,766 |
| ENV_8436_8443 | chrX:92578106-92579623 | RS6615516 | 92571311 | 0,728 |
| ENV_8436_8443 | chrX:92578106-92579623 | RS6619304 | 92572635 | 0,75 |
| ENV_8436_8443 | chrX:92578106-92579623 | RS6619307 | 92573053 | 0,697 |
| ENV_8436_8443 | chrX:92578106-92579623 | RS5940190 | 92575134 | 0,853 |
| ENV_8436_8443 | chrX:92578106-92579623 | RS5983521 | 92580150 | 0,908 |
| ENV_8436_8443 | chrX:92578106-92579623 | RS5983522 | 92580206 | 0,974 |
|  |  | rs1332885 |  | 0,335 |
|  |  | rs318173 |  | 0,87 |
| POL_8943 | chrX:96985401-96987847 | RS2125324 | 96975499 | 0,867 |
|  |  | rs318190 | #REFERENCE! | 0,696 |
|  |  | rs618561 | #REFERENCE! | 0,841 |
|  |  | rs387312 | #REFERENCE! | 0,001 |
| POL_8943 | chrX:96985401-96987847 | RS400586 | 96980522 | <0,001 |
| POL_8943 | chrX:96985401-96987847 | RS391745 | 96981136 | <0,001 |
| POL_8943 | chrX:96985401-96987847 | RS11797742 | 96981356 | 0,011 |
| POL_8943 | chrX:96985401-96987847 | RS421531 | 96981831 | 0,991 |
|  |  | RS628526 | 96983253 | 0,399 |
| POL_8943 | chrX:96985401-96987847 | RS445913 | 96983701 | 0,956 |
|  |  | RS440200 | 96983753 |  |
| POL_8943 | chrX:96985401-96987847 | RS11092162 | 96984819 | 0,06 |
|  |  | RS11092163 | 96984854 | 0,039 |
| POL_8943 | chrX:96985401-96987847 | RS318138 | 96984891 | <0,001 |
|  |  | RS318137 | 96984986 | 0,541 |
| POL_8943 | chrX:96985401-96987847 | RS6620396 | 96985496 | 0,047 |
| POL_8943 | chrX:96985401-96987847 | RS6620397 | 96985527 | 0,004 |
| POL_8943 | chrX:96985401-96987847 | RS318136 | 96985785 | 0,497 |
| POL_8943 | chrX:96985401-96987847 | RS318134 | 96985802 | 0,939 |
|  |  | RS318133 | 96986762 | 0,946 |
|  |  | RS12845339 | 96986910 | 0,462 |
|  |  | RS6615948 | 96986949 | 0,009 |
| POL_8943 | chrX:96985401-96987847 | RS318132 | 96987014 | 0,919 |
| POL_8943 | chrX:96985401-96987847 | RS5920968 | 96987505 | 0,172 |
|  |  | rs 5920969 | #REFERENCE! | 0,018 |
| POL_8943 | chrX:96985401-96987847 | RS318131 | 96988745 | 0,001 |
|  |  | RS 16982583 | 96989159 |  |
| POL_8943 | chrX:96985401-96987847 | RS4827909 | 96989280 | 0,007 |
|  |  | rs71534260 | #REFERENCE! | 0,419 |
| POL_8943 | chrX:96985401-96987847 | RS6620400 | 96989621 | 0,731 |
|  |  | RS17333695 | 96990118 | 0,004 |
| POL_8943 | chrX:96985401-96987847 | RS318129 | 96991896 | 0,002 |
| POL_8943 | chrX:96985401-96987847 | RS2379168 | 96994230 | 0,758 |
| POL_8943 | chrX:96985401-96987847 | RS318156 | 96994406 | 0,429 |
| POL_8943 | chrX:96985401-96987847 | RS167609 | 96995093 | 0,815 |
| POL_8943 | chrX:96985401-96987847 | RS318157 | 96995537 | 0,852 |
| POL_8943 | chrX:96985401-96987847 | RS16982595 | 96996337 | 0,253 |
| POL_8943 | chrX:96985401-96987847 | RS170320 | 96997113 | 0,878 |
| POL_8943 | chrX:96985401-96987847 | RS675270 | 96997562 | 0,691 |
|  |  | rs318190 | #REFERENCE! | 0,696 |
|  |  | rs618561 | #REFERENCE! | 0,841 |
|  |  | rs387312 | #REFERENCE! | 0,001 |
| ENV_8944 | chrX:96988853-96990668 | RS400586 | 96980522 | <0,001 |
| ENV_8944 | chrX:96988853-96990668 | RS391745 | 96981136 | <0,001 |
| ENV_8944 | chrX:96988853-96990668 | RS11797742 | 96981356 | 0,011 |
| ENV_8944 | chrX:96988853-96990668 | RS421531 | 96981831 | 0,991 |
|  |  | RS628526 | 96983253 | 0,399 |
| ENV_8944 | chrX:96988853-96990668 | RS445913 | 96983701 | 0,956 |
| ENV_8944 | chrX:96988853-96990668 | RS11092162 | 96984819 | 0,06 |
|  |  | RS11092163 | 96984854 | 0,039 |
| ENV_8944 | chrX:96988853-96990668 | RS318138 | 96984891 | <0,001 |
| ENV_8944 | chrX:96988853-96990668 | RS6620396 | 96985496 | 0,047 |
| ENV_8944 | chrX:96988853-96990668 | RS6620397 | 96985527 | 0,004 |
| ENV_8944 | chrX:96988853-96990668 | RS318136 | 96985785 | 0,497 |
| ENV_8944 | chrX:96988853-96990668 | RS318134 | 96985802 |  |
|  |  | RS318133 | 96986762 | 0,946 |
|  |  | RS12845339 | 96986910 | 0,462 |
|  |  | RS6615948 | 96986949 | 0,009 |
| ENV_8944 | chrX:96988853-96990668 | RS318132 | 96987014 | 0,919 |
| ENV_8944 | chrX:96988853-96990668 | RS5920968 | 96987505 | 0,172 |
|  |  | rs 5920969 | #REFERENCE! | 0,018 |
| ENV_8944 | chrX:96988853-96990668 | RS318131 | 96988745 | 0,001 |
|  |  | RS16982583 | 96989159 |  |
| ENV_8944 | chrX:96988853-96990668 | RS4827909 | 96989280 | 0,007 |
|  |  | rs71534260 | #REFERENCE! | 0,419 |
| ENV_8944 | chrX:96988853-96990668 | RS6620400 | 96989621 | 0,731 |
|  |  | RS17333695 | 96990118 | 0,004 |
| ENV_8944 | chrX:96988853-96990668 | RS318129 | 96991896 | 0,002 |
| ENV_8944 | chrX:96988853-96990668 | RS2379168 | 96994230 | 0,758 |
| ENV_8944 | chrX:96988853-96990668 | RS318156 | 96994406 | 0,429 |
| ENV_8944 | chrX:96988853-96990668 | RS167609 | 96995093 | 0,815 |
| ENV_8944 | chrX:96988853-96990668 | RS318157 | 96995537 | 0,852 |
| ENV_8944 | chrX:96988853-96990668 | RS16982595 | 96996337 | 0,253 |
| ENV_8944 | chrX:96988853-96990668 | RS170320 | 96997113 | 0,878 |
| ENV_8944 | chrX:96988853-96990668 | RS675270 | 96997562 | 0,691 |
| ENV_8944 | chrX:96988853-96990668 | RS318166 | 97000485 | 0,864 |
|  |  | rs318168 |  | 0,774 |
|  |  | rs1332886 |  | 0,337 |
|  |  | rs318158 |  | 0,627 |
|  |  | rs318162 |  | 0,737 |
| ENV_24244_24243 | chrX:104798132-104799657 | RS7891183 | 104789930 |  |
| ENV_24244_24243 | chrX:104798132-104799657 | RS5916942 | 104790537 | 0,606 |
| ENV_24244_24243 | chrX:104798132-104799657 | RS5916943 | 104796734 | 0,551 |
| ENV_24413 | chrX:106182394-106183903 | RS5962376 | 106179619 | 0,599 |
| ENV_24413 | chrX:106182394-106183903 | RS1152324 | 106180145 | 0,616 |
| ENV_24413 | chrX:106182394-106183903 | RS5962377 | 106186477 | 0,735 |
| ENV_24413 | chrX:106182394-106183903 | RS1152326 | 106187762 | 0,878 |
| ENV_27933_27932 | chrX:148565692-148567722 | RS9698929/rs6540338 | 148571890 | 0,448 |
| ENV_27933_27932 | chrX:148565692-148567722 | RS9699115/rs6419443 | 148572235 | 0,418 |
| ENV_27933_27932 | chrX:148565692-148567722 | RS6419444 | 148573723 | 0,573 |
