# Supplementary material for: The Etiology of Multiple Sclerosis: Genetic Evidence for the Involvement of the Human Endogenous Retrovirus HERV-Fc1
Source: PLoS One. 2011 Feb 2;6(2):e16652. doi: 10.1371/journal.pone.0016652 (PMC3032779; doi:10.1371/journal.pone.0016652)
Supplement: Supplementary file 6 [file pone.0016652.s006.doc]

Cohort	Sex	Status	     Genotype for rs391745	P(C-allele)	Odds Ratio (CI95)	P-values		 	 	 	C/CC	CG	G/GG	 	for alleles	 		2
 
 
 
 	Male	Cases	18	0	105	0.146	3.73(2.86-4.60)	0.0028			 	Controls	8	0	174	0.044	 	 			Female	Cases	4	57	141	0.161	2.03 (1.66 - 2.40)	1.9*10-4			 	Controls	4	56	311	0.086	 	 			Sexes combined	 	 	 	 	 	2.26 (1.87 - 2.54)	2.3*10-6		3
 
 
 
 	Male	Cases	22	0	141	0.135	1.92 (1.34 - 2.50)	0.036			 	Controls	31	0	382	0.075	 	 			Female	Cases	1	76	266	0.113	1.21 (0.91 - 1.50)	0.22			 	Controls	5	129	590	0.096	 	 			Sexes combined	 	 	 	 	 	1.31 (1.07 - 1.59)	0.014		4
 
 
 
 	Males	Cases	5	0	65	0.071	0.67 (0 - 1.72)	0.62			 	Controls	15	0	131	0.103	 	 			Females	Cases	2	28	131	0.099	1.12 (0.64 - 1.60)	0.62			 	Controls	0	44	202	0.089	 	 			Sexes combined	 	 	 	 	 	1.02 (0.59 - 1.45)	0.66		2+3+4
 
 
 
 	Male	Cases	45	0	310	0.127	1.85(1.47 - 2.31)	4*10-5			 	Controls	54	0	687	0.073	 	 			Female	Cases	7	161	539	0.124	1.38 (1.19 - 1.60)	0.003			 	Controls	9	185	901	0.093	 	 			Sexes combined	 	 	 	 	 	1.47 (1.29 - 1.66)	6*10-7
